# Preventive and Therapeutic Effects of Plant‐Derived Compounds on Tooth Erosion: A Systematic Review and Meta‐Analysis of *In Situ* and *In Vitro* Studies

**DOI:** 10.1002/cre2.70235

**Published:** 2025-10-28

**Authors:** Mahtab Memarpour, Neda Afzali Baghdadabadi, Golnoush Farzinnia, Mahya Agharokh, Niloofar Mokhtari, Erfan Bardideh

**Affiliations:** ^1^ Oral and Dental Disease Research Center, School of Dentistry Shiraz University of Medical Sciences Shiraz Iran; ^2^ College of Dentistry University of Saskatchewan Saskatoon Saskatchewan Canada; ^3^ School of Dentistry Shiraz University of Medical Sciences Shiraz Iran; ^4^ Dental Research Center Mashhad University of Medical Sciences Mashhad Iran

**Keywords:** plant extract, tooth erosion, tooth hardness, tooth roughness

## Abstract

**Objectives:**

Tooth erosion is the nonbacterial and irreversible pathological dissolution of enamel and dentin, and is primarily influenced by lifestyle. This systematic review and meta‐analysis aimed to evaluate the preventive and therapeutic effects of plant‐derived compounds on tooth erosion using evidence from *in vitro* and *in situ* studies.

**Materials and Methods:**

The review was registered in PROSPERO (CRD42023493906) and search was performed in PubMed, Scopus, ISI Web of Science, and Embase databases until July 5, 2025. *In vitro* and *in situ* original studies that investigated the effects of plant‐based interventions on dental erosion, with a control group, were included. *In vivo* studies and those plants combined with non‐plant substances were excluded. Risk of bias was assessed using the QUIN tool. Meta‐analyses were performed when appropriate. Mechanical and ultrastructure assessments used to evaluate surface loss, hardness, and morphological changes were also reviewed.

**Results:**

Out of 1119 studies, 38 studies met the inclusion criteria; 22 studies assessed preventive effects, and 18 assessed the therapeutic effects, and one study that evaluated both the effects. Most studies had a medium risk of bias. Meta‐analysis showed that quercetin as a preventive measure, significantly reduced dentin loss by 4.95 µm with high heterogeneity (*I* ^2^ = 98%). For therapeutic measures, green tea reduced dentin loss by 0.89 µm, whereas epigallocatechin‐3‐gallate (EGCG) did not significantly decrease dentin loss. The two analyses were heterogeneous (*I* ^2^ = 99%). Quercetin significantly decreased dentin loss by 4.19 µm with low heterogeneity (*I* ^2^ = 0%).

**Conclusions:**

Plants with polyphenols may modify the pellicle layer ultrastructure to protect teeth from erosion. Quercetin (the most common flavonoid in fruit and vegetables) has preventive and therapeutic effects on dentin erosion. Green tea (because of its elevated catechin content) has therapeutic effects on dentin erosion.

## 1 Introduction

Tooth erosion is an irreversible pathological dissolution of enamel and dentin of primary or permanent teeth due to acidic chemical processes that are not caused by bacterial agents and dental plaque (Warreth et al. 2020). The mean erosion prevalence is approximately 30%–50% in primary and 20%–45% in permanent dentitions (Schlueter and Luka 2018). Erosion is usually slow and asymptomatic; therefore, patients do not seek treatment until the onset of problems.

Erosion is a multifactorial process caused by internal or external factors. Internal factors include gastroesophageal reflux such as gastritis and hiatus hernia caused by alcohol, pregnancy, obesity, and eating disorders (bulimia, anorexia nervosa) (Warreth et al. 2020; Levrini et al. 2014). External factors include acidic and carbonated drinks, energy drinks, citrus fruits, fruit juices, pickles, and medicines such as effervescent tablets. Work environments such as swimming pools that use a weaker chlorine to disinfect water or battery factories where workers are exposed to vaporized sulfuric acid are other external sources. Decreased saliva flow after surgical removal of major salivary glands, Sjögren's syndrome, drug use (antidepressants and sedatives) or head and neck radiotherapy predisposes individuals to tooth erosion (Warreth et al. 2020; Zebrauskas et al. 2014). The severity of the erosion depends on the type and temperature of the acid, pH, concentration, and duration of tooth exposure (Barbour and Rees 2006). Erosion is a dynamic process that begins with enamel demineralization, and results in decreased hardness and increased roughness. Dentin is more susceptible to surface loss than enamel because of its higher organic matrix content and different crystalline forms. Loss of peritubular and intertubular dentin exposes their minerals and the organic matrix fibers. The changes in the dentin fluid flow may also cause tooth hypersensitivity that plays a role in poor oral health and esthetics (Levrini et al. 2014; Ganss et al. 2014).

Various chemical and bioactive products have been developed to reduce the problems attributed to erosion. Bioactive agents improve resistance to biodegradation and demineralization of the dentin collagen matrix (Aguiar et al. 2014; Broyles et al. 2013; Dos Santos et al. 2011; Liu et al. 2013). Leaves, fruit, or roots and other plant‐derived compounds are potential sources of new pharmacologically bioactive agents that may help protect against dental erosion (Weber et al. 2015; Niemeyer et al. 2023a, 2021; Hertel et al. 2016; Schestakow et al. 2022; Zhao et al. 2025). Although the public has a positive opinion of plant‐derived compounds (Tzimas et al. 2024), there is a lack of information on their preventive and therapeutic uses for tooth erosion. The aim of this systematic review and meta‐analysis is to assess the preventive and therapeutic effects of plant‐derived compounds on tooth erosion as reported by *in vitro* and *in situ* studies.

## 2 Methods

### 2.1 Registration

We conducted this systematic review of *in vitro* and *in situ* studies in accordance with the PRISMA 2020 recommendations. The review is registered in the International Prospective Register of Systematic Reviews for English and Persian papers (CRD42023493906). However, only English‐language statistical data were ultimately included due to a lack of Persian studies that did not meet the inclusion criteria. In addition to tea, other plant extracts with potential anti‐erosive effects were also assessed in this review.

We based this review on the PICO model of population (P): human or bovine enamel or dentin; intervention (I): application of different plant extracts before or after erosive challenge; control (C): water, distilled and/or deionized water, and no treatment; and outcome (O): qualitative or quantitative data about the surface hardness, surface roughness, surface loss, pellicle ultrastructure, and surface morphological changes.

### 2.2 Eligibility Criteria

Inclusion criteria consisted of:

1. *In vitro* and *in situ* studies.
2. Studies that published statistical data in English, which assessed human or bovine enamel and dentin.
3. Studies that analyzed the effect of plant (or herbal) extracts on erosion of enamel and dentin specimens.
4. Studies that measured the preventive and/or therapeutic effect of plant extracts on teeth. Studies classified as preventive focused on interventions applied before erosive challenges and aimed to prevent or reduce initial mineral loss in enamel or dentin. In contrast, studies categorized as therapeutic involved interventions applied after erosive damage, which intended to repair, reinforce, or minimize further tissue loss.
5. Studies that evaluated surface hardness, surface roughness, surface loss, pellicle ultrastructure, and surface morphological changes.
6. Studies that included a control group of deionized or distilled water, water, or no treatment.
7. Enamel and dentin specimens that were subjected to erosive/acidic challenges.

The following articles were excluded: (1) *in vivo* studies; (2) studies of plants combined with non‐plant substances such as fluoride, honey, or chocolate; (3) studies that lacked a comparison between experimental plant and control groups; (4) animal studies; or (5) case reports, case series, observational studies, randomized controlled trials, clinical trials, congress and review articles, abstracts, interviews, editorials, or opinions.

### 2.3 Information Source and Search Strategy

PubMed (Medline, PMC), Scopus, ISI Web of Science, and Embase were searched until July 5, 2025 using a combination of relevant keywords (Table 1). In addition, the reference lists of the indicated articles were manually searched to identify other related studies. Grey literature was not included due to inconsistent reporting standards and limited applicability to *in vitro*/*in situ* methodologies.

**Table 1:** Search history.

PubMed (Medline/PMC)	
#1	((((“tooth erosion”) OR (“enamel erosion”)) OR (“dentin erosion”)) OR (“dental erosion”)) OR (“erosive wear”)
#2	((((((((((((tea) OR (“plant extract”)) OR (“plant oil”)) OR (“plant*”)) OR (“leaves extract”)) OR (“seed extract”)) OR (“herbal”)) OR (“polyphenol*”)) OR (“epigallocatechin gallate”)) OR (“*camellia sinensis*”)) OR (“flavonoid*”)) OR (proanthocyanidin)) or (quercetin)
#3	Search: (#1) AND (#2)
**Scopus**	
#1	(TITLE‐ABS‐KEY (dental erosion) OR TITLE‐ABS‐KEY (tooth erosion) OR TITLE‐ABS‐KEY (enamel erosion) OR TITLE‐ABS‐KEY (dentin erosion) OR TITLE‐ABS‐KEY (erosive wear))
#2	(TITLE‐ABS‐KEY (tea) OR TITLE‐ABS‐KEY (“plant extract”) OR TITLE‐ABS‐KEY (“plant oil”) OR TITLE‐ABS‐KEY (plant) OR TITLE‐ABS‐KEY (“leaves extract”) OR TITLE‐ABS‐KEY (“seed extract”) OR TITLE‐ABS‐KEY (herbal) OR TITLE‐ABS‐KEY (polyphenol) OR TITLE‐ABS‐KEY (epigallocatechin gallate”) OR TITLE‐ABS‐KEY (“*camellia sinensis*”) OR TITLE‐ABS‐KEY (flavonoid) OR TITLE‐ABS‐KEY (proanthocyanidin) OR TITLE‐ABS‐KEY (quercetin)
#3	Search: (#1) AND (#2)
**Web of science core collection**	
#1	“dental erosion” (All Fields) or “tooth erosion” (All Fields) or “enamel erosion” (All Fields) or “dentin erosion” (All Fields) or “erosive wear” (All Fields)
#2	tea (All Fields) or “plant extract” (All Fields) or “plant oil” (All Fields) or plant (All Fields) or “leaves extract” (All Fields) or “seed extract” (All Fields) or herbal (All Fields) or polyphenols (All Fields) or “epigallocatechin gallate” (All Fields) or “*Camellia sinensis*” (All Fields) or flavonoid (All Fields) or proanthocyanidin (All Fields) or quercetin (All Fields)
#3	Search: (#1) AND (#2)
**Embase**	
#1	(‘dental erosion’ OR ‘tooth erosion’ OR ‘enamel erosion’ OR ‘dentin erosion’ OR ‘erosive wear’)
#2	(tea OR ‘plant extract’ OR ‘plant oil’ OR ‘vegetable oil’ OR ‘plant’ OR ‘leaves extract’ OR ‘seed extract’ OR ‘polyphenol’ OR ‘herbal’ OR ‘epigallocatechin gallate’ OR ‘*camellia sinensis*’ OR ‘flavonoid’ OR ‘proanthocyanidin’ OR ‘quercetin’)
#3	Search: (#1) AND (#2)

### 2.4 Study Selection and Data Extraction

Two reviewers (F.G.; A.N.) independently performed study selection and data extraction. Disagreements were discussed and resolved by a third independent expert (M.M.). Duplicate studies were removed and the remaining titles and abstracts were assessed according to the eligibility criteria. Next, full texts of potentially eligible studies were reviewed by all of the authors. For each study, the following data were extracted: author's name, year of publication, study type, methodology (type of specimen, plant extract, and control group), evaluation method (qualitative or quantitative regarding the surface hardness, roughness, surface loss, pellicle ultrastructure, and surface morphological changes), and main results. In addition, the reference lists of the indicated articles were manually searched to locate other related studies. In cases where methods or results in the included studies were unclear or incomplete, we tried to contact the corresponding authors for three times to obtain clarification or additional data. If no response was received, we noted the missing information and proceeded with the available data without making unverified assumptions.

### 2.5 Assessment of Risk of Bias and Quality of Evidence

The reviewers (A.N.; A.M.; M.N.) independently evaluated the risk of bias in the selected studies by using the Quality Assessment Tool for In Vitro Studies (QUIN tool) (Sheth et al. 2024). The QUIN tool has 12 criteria: clearly stated aims/objectives, detailed explanation of sample size calculation, sampling technique, comparison group, methodology, operator details, randomization, method of measurement of outcome, outcome assessor details, blinding, statistical analysis, and presentation of results. The researchers assigned scores of two points for each criterion if adequately specified, one point if the criteria were inadequately specified, and zero points if not specified in the article. The reviewers resolved any disagreements via consensus before assigning the final score for the risk of bias. The scores were then added to obtain a total score for each study, and the final score was obtained by using the following formula:

Finalscore=(Totalscore×100)/(2×numberofcriteriaapplicable)

The final scores were used to grade the study as high (< 50%), medium (50% to 70%), or low risk (> 70%).

### 2.6 Data Synthesis

Only the preventive and therapeutic effects of solutions for dentin loss were evaluated in the meta‐analysis because of the lack of homogeneous studies. Because of the continuous nature of the outcome, mean differences for the continuous outcome were calculated using inverse variance meta‐analysis with random‐effect models. Study heterogeneity was assessed using the chi‐squared *Q* test and the *I*² metric. All meta‐analyses were conducted by B.E. with STATA software (version 17, StataCorp. LLC). A 95% confidence interval (CI) was used, and *p*‐values less than 0.05 were considered statistically significant. The publication bias could not be assessed because each analysis included less than 10 studies.

## 3 Results

### 3.1 Study Selection

**Figure 1:**
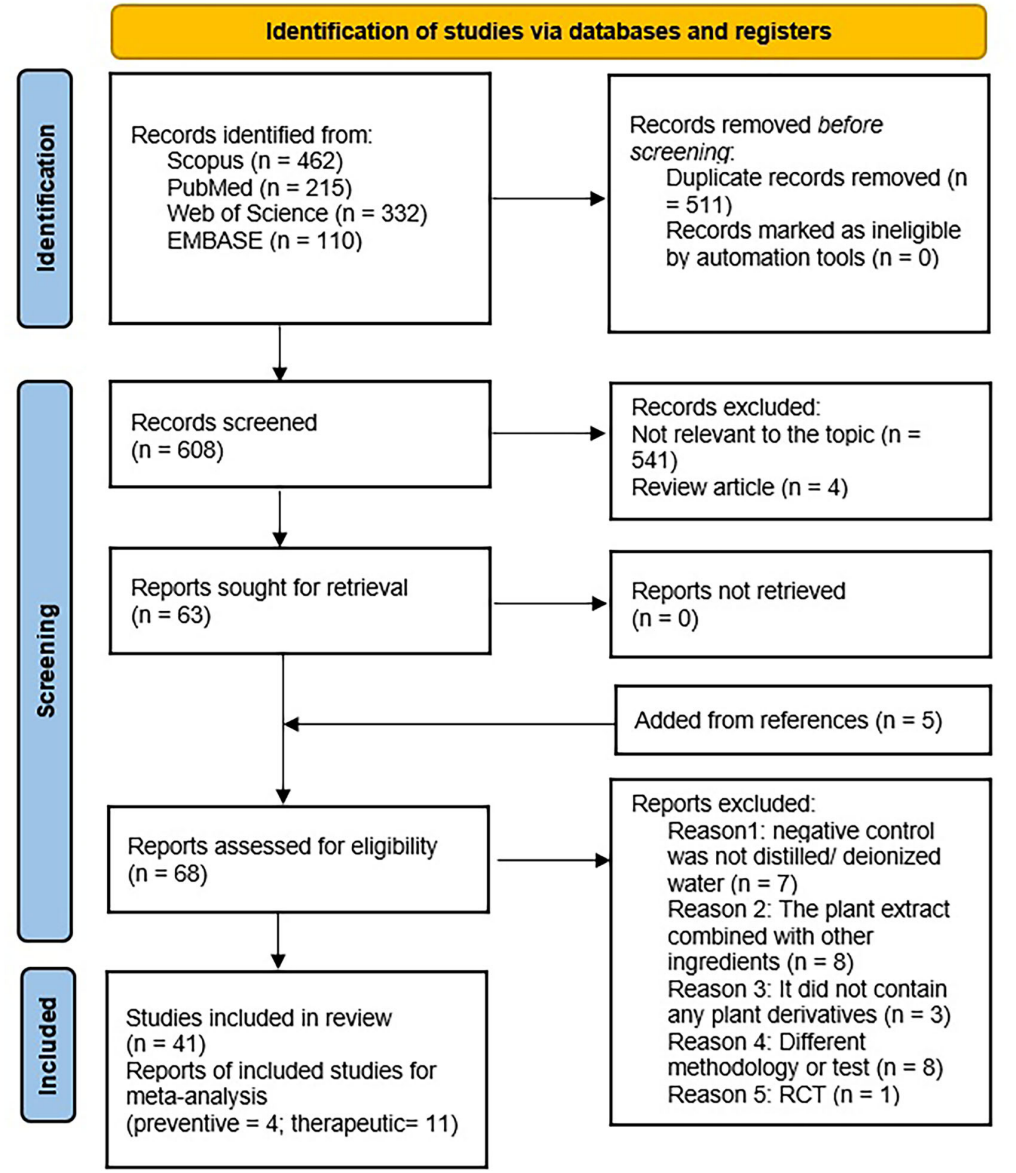
Prisma flow diagram of the study.

Figure 1 summarizes the selection process according to PRISMA. A search from all databases resulted in 1119 potential articles. We eliminated all duplicate and irrelevant articles, and screened the remaining 63 articles. The hand searches revealed five more article for screening. From these 68 articles, 27 did not meet the eligibility criteria and were excluded (Table S1).

### 3.2 Study Characteristics

We included 41 studies in the final analysis, 33 *in vitro* and 8 *in situ* designs. Most focused on polyphenol‐rich plant extracts such as green tea (*Camellia sinensis*), grape seed (*Vitis vinifera*), cranberry, and other. Control groups commonly used deionized or distilled water.

A total of 23 studies evaluated preventive effects (Weber et al. 2015; Niemeyer et al. 2021; Hertel et al. 2016; Schestakow et al. 2022, 2024; Baumann et al. 2023; Niemeyer et al. 2023b; Hong et al. 2022; Capalbo et al. 2022; Iftikhar et al. 2022; Rios et al. 2021; Sarialioglu Gungor and Donmez 2021; Jiang et al. 2020a; Ionta et al. 2018; Wang et al. 2018; Hertel et al. 2017; Ionta et al. 2017; Sales‐Peres et al. 2016; Hannig et al. 2012; Guo et al. 2012; Souza et al. 2010; Wiegand et al. 2007; Li et al. 2022), 19 studies evaluated therapeutic effects (Niemeyer et al. 2023a; Li et al. 2022; Martins et al. 2024; Obeid et al. 2023; Rabelo et al. 2023; Kato et al. 2022; Carvalho et al. 2022; Celik et al. 2021; DE Moraes et al. 2021; Cardoso et al. 2020; Jiang et al. 2020b; Ozan et al. 2020; Passos et al. 2018; De Moraes et al. 2016; Silveira et al. 2014; Mirkarimi and Toomarian 2012; Zhang et al. 2009; Kato et al. 2009; Magalhães et al. 2009). One study evaluated both preventive and therapeutic effects of plant extracts on tooth erosion (Li et al. 2022). Tests used to evaluate surface loss or surface hardness included: profilometry, Knoop, Vickers, atomic force microscopy, and laser scanning confocal microscopy. Scanning electron microscopy and transmission electron microscopy were used to evaluate surface morphological changes or pellicle ultrastructure. Tables 2 and 3 show the methodological characteristics of the studies.

**Table 2:** Preventive effect of plant extract application before the erosive challenge.

Author's name	Study type	Specimen	Plant extract	Control	Evaluated main parameters (method)	Outcome
Schestakow et al. (2024)	*In situ*	Bovine dentin	a. 1% tannic acid b. 1% hop	Sterile water	Pellicle's ultrastructure (TEM)	Thicker, more electron‐dense pellicle after application of the polyphenol tannic acid or, to a lesser extent, the polyphenolic hop extract.
Baumann et al. (2023)	*In vitro* (human saliva)	Human enamel	a. Grape seed b. Cranberry	Deionized water	Surface microhardness (Knoop hardness test)	Both plant extracts: No difference (*p* > 0.05). Although the extracts alone showed no effect, their combination with fluoride led to a significant protection.
Niemeyer et al. (2023b)	*In vitro* (human saliva)	Human dentin	a. Green tea b. Blueberry c. Grape seed	Deionized water	Surface loss (profilometry)	Non‐pellicle subgroups: All plant extracts showed no difference (*p* > 0.05). Pellicle subgroups: Only BE provided protection.
Hong et al. (2022)	*In situ*	Human dentin	Quercetin (Q75, Q150, and Q300)	Deionized water	Profilometry	Compared to the negative controls, all treatment solutions significantly reduced dentin loss.
Schestakow et al. (2022)	*In situ*	Bovine enamel	a. Black tea b. Tannic acid	No treatment	Pellicle's ultrastructure (TEM)	Consumption of polyphenolic beverages enhanced the anti‐erosive potential of the enamel pellicle. There was an increase in pellicle thickness and density after treatment with polyphenols.
Capalbo et al. (2022)	*In vitro*	Bovine dentin	Quercetin	Placebo	1. Surface hardness (Knoop hardness test) 2. Surface loss (profilometry) 3. Surface morphological changes (SEM)	1. Increased (*p* < 0.001). 2. Decreased (*p* < 0.001). 3. SEM: Partial obliteration was observed for the group treated with quercetin.
Iftikhar et al. (2022)	*In vitro*	Human enamel	EGCG	Distilled water	1. Diameter of dentinal tubule orifices (SEM) 2. Microhardness (HMV‐2 tester)	1. Decreased (*p* < 0.001).Surface roughness of the enamel was greater in the control group compared to the EGCG group. 2. Increased (*p* = 0.002).
Li et al. (2022)	*In vitro*	Human dentin	Quercetin	Deionized water	1. Surface loss (profilometry) 2. Surface morphological changes (SEM)	1. Decreased (*p* < 0.001).The preventive effect was better than the treatment effect (*p* < 0.001). 2. For quercetin, a greater level of occlusion or narrowing of the dentinal tubules was found before the erosive challenges.
Rios et al. (2021)	*In vitro*	Bovine enamel	Palm oil	Deionized water	Surface hardness loss (Knoop hardness test)	Decreased (*p* < 0.05).
Niemeyer et al. (2021)	*In vitro* (human saliva)	Human enamel	a. Green tea b. Black tea c. Peppermint tea d. Rosehip tea e. Grape seed f. Grapefruit seed g. Cranberry h. Propolis	Deionized water	Surface microhardness (Knoop hardness test)	‐ Green tea and black tea: Increased (*p* < 0.05). There was no significant difference between the green tea and black tea groups. Peppermint tea: No difference (*p* > 0.05). ‐ Rosehip tea was very erosive and the final surface microhardness could not be measured as it was below the detection limit. Therefore, it was not included in the statistical analysis. Grape seed and grapefruit seed: Increased (*p* < 0.05). ‐ Grape seed extracts significantly performed better than grapefruit seed extracts. ‐ Cranberry: Decreased (*p* < 0.05). ‐ Propolis: No difference.
Sarialioglu Gungor and Donmez (2021)	*In vitro*	Bovine dentin	a. Green tea b. Rosehip c. Clove d. Pomegranate e. Grape seed	Fluoride ion‐free water	1. Nanohardness (nanoindentation) 2. Surface roughness (AFM) 3. Surface morphological changes (SEM)	1. Clove and green tea: Increased (*p* < 0.05). There was no significant difference between clove and green tea extracts. Pomegranate and grape seed: No difference (*p* > 0.05). Rosehip: Decreased. Grape seed significantly decreased nanohardness more than rosehip. 2. All plant extracts: No difference. 3. Macromolecular deposits were observed both in the SEM and AFM images of the pomegranate, colgate, and rosehip groups. It can be concluded that the green tea and clove extract groups were more successful in preventing dentin erosion than the other groups.
Jiang et al. (2020a)	*In vitro*	Human dentin	a. Epigallocatechin gallate b. 0.075, 0.150, and 0.300 mg/L quercetin	Deionized water	1. Profilometry 2. Surface morphological changes (SEM)	Immersion in the quercetin solution is effective in improving the dentin resistance erosion
Ionta et al. (2018)	*In situ*	Bovine enamel	Palm oil	Deionized water	Surface loss (profilometry)	Decreased (*p* < 0.05).
Wang et al. (2018)	*In vitro*	Human enamel and dentin	EGCG	Distilled water	1. Surface roughness (LSCM) 2. Surface hardness loss (HMV‐2 tester) 3. Surface morphological changes (SEM)	1. Enamel: Decreased (*p* < 0.05); Dentin: No difference 2. Decreased (*p* < 0.05) 3. More erosive damage was present in the eroded area of the control group.
Hertel et al. (2017)	*In situ*	Bovin enamel	Tannic acid	No treatment	Pellicle's ultrastructure (TEM)	TEM imaging indicated that rinsing with tannic acid yielded a sustainable modification of the pellicle, which was distinctly more electron‐dense.
Ionta et al. (2017)	*In situ*	Bovine enamel	a. Palm oil b. Coconut oil c. Safflower oil d. Sunflower oil e. Olive oil	Deionized water	Surface hardness loss (Knoop hardness test)	Pure palm oil: Decreased (*p* < 0.05). Other oils: No difference (*p* > 0.05). There was no significant difference between all vegetable oils.
Hertel et al. (2016)	*In situ*	Bovine enamel	*Inula viscosa* tea	No treatment	Pellicle's ultrastructure (TEM)	TEM investigation indicated a modification of the pellicle's ultrastructure, but no enhanced protection against erosive noxae.
Sales‐Peres et al. (2016)	*In situ*	Human enamel/dentin	*Euclea natalensis*	No treatment	Surface loss (profilometry)	Enamel: No difference (*p* > 0.05). Dentin: Decreased (*p* = 0.001).
Weber et al. (2015)	*In situ*	Bovine enamel	a. *Ribes nigrum* leaves b. *Origanum vulgare* c. *Ribes nigrum* leaves and oregano	No treatment	Pellicle's ultrastructure (TEM)	Rinsing with *Ribes nigrum* leaves/*Origanum vulgare* yielded a distinctly thicker and more electron‐dense pellicle.
Hannig et al. (2012)	*In situ*	Bovine enamel	Safflower oil	No treatment	Pellicle's ultrastructure (TEM)	The rinses with the edible oil had no protective effects on the degradation of the pellicle layer. The degradation process of the proteinaceous layer occasionally seemed to be more pronounced in the oil‐rinsed specimens.
Guo et al. (2012)	*In vitro*	Bovine dentin	Galla chinensis	Deionized water	Laser scanning confocal microscopy (LSCM)	Galla chinensis enhanced the remineralization of artificial root lesion.
Souza et al. (2010)	*In vitro*	Bovine enamel	a. 10% xylitol solution b. 20% xylitol solution	No treatment	1. Surface loss (profilometry) 2. Surface morphological changes (SEM)	1. Both xylitol solutions: Decreased (*p* < 0.05) 2. SEM images of the eroded specimens untreated and retreated with 20% xylitol solution showed a demineralized surface. 10% xylitol solution, showed smoother layer in comparison to the other group.
Wiegand et al. (2007)	*In vitro*	Bovine enamel/dentin	a. Pure olive oil b. 2% olive oil	Distilled water	Surface loss (profilometry)	Pure olive oil: No difference. 2% olive oil: Enamel loss: Decreased; Dentin loss: No difference.

Abbreviations: AFM, atomic force microscopy; EGCG, epigallocatechin‐3‐gallate; LSCM, laser scanning confocal microscopy; SEM, scanning electron microscopy; TEM, transmission electron microscopy.

**Table 3:** Therapeutic effect of plant extract application after the erosive challenge.

Author's name	Study type	Specimen	Plant extract	Control	Evaluated main parameters (method)	Outcome
Martins et al. (2024)	*In situ*	Bovine enamel	a. Palm oil b. 2% Proanthocyanidin c. 2% Proanthocyanidin + palm oil	Deionized water	Surface loss (profilometry)	Decreased for palm oil and proanthocyanidin groups (*p* < 0.05).
Niemeyer et al. (2023a)	*In vitro* (human saliva)	Human dentin	a. Açaí extract b. Blueberry extract c. Green tea extract d. Grape seed extract	Deionized water	Surface loss (profilometry)	Açaí extract and blueberry extracts: No difference (*p* > 0.05). Green tea and grape seed extracts: Decreased (*p* < 0.0001). There was no significant difference between the green tea and grape seed extract groups.
Obeid et al. (2023)	*In vitro*	Human dentin and cementum	a. 50 mg/mL *Moringa oleifera* leaves (M1) b. 200 mg/mL *Moringa oleifera* leaves (M2)	No treatment	Surface morphological changes (SEM)	*Moringa oleifera* leaves had an extraordinary effect on the remineralization of coronal dentin and acellular cementum.
Rabelo et al. (2023)	*In vitro*	Human dentin	a. 0.5% Juca seed galactomannan b. 1% Juca seed galactomannan	Distilled water	1. Surface loss (profilometry) 2. Surface morphological changes (SEM)	1. Both plant extracts: No difference (*p* > 0.05) 2. SEM images: The samples treated with 0.5% Juca seed galactomannan displayed the formation of poorly defined crystals, while samples treated with 1% Juca seed galactomannan showed a higher quantity of crystals with a more distinct shape.
Carvalho et al. (2022)	*In vitro* (human saliva)	Human enamel	a. Grape seed extract b. Grapefruit seed extract c. Blueberry extract	Deionized water	Surface hardness (Vickers hardness test)	All plant extracts without fluoride: No difference (*p* > 0.05). The presence of fluoride provided better protection than the groups that contained extract or fluoride only. Grape seed extract showed the best protection.
Kato et al. (2022)	*In vitro*	Bovine dentin	a. Green tea extract solution b. Cranberry extract	Distilled water	Surface loss (profilometry)	Both plant extracts: Decreased (*p* < 0.05). There was no significant difference between the treatment groups.
Li et al. (2022)	*In vitro*	Human dentin	Quercetin	Deionized water	1. Surface loss (profilometry) 2. Surface morphological changes (SEM)	1. Decreased (*p* < 0.001). 2. A greater level of occlusion or narrowing of the dentinal tubules was found in the pre subgroup compared to the post subgroup.
Celik et al. (2021)	*In vitro*	Human enamel	Ginger	Deionized water	Surface microhardness (Vickers hardness test)	No difference (*p* = 0.664).
DE Moraes et al. (2021)	*In situ*	Human dentin	a. EGCG b. Green tea	No treatment	1. Surface roughness (profilometry) 2. Surface loss (profilometry) 3. Surface morphological changes (SEM)	1. Both plant extracts: No difference (*p* > 0.05). 2. Both plant extracts: No difference (*p* > 0.05). 3. Based on SEM analysis, the green tea solution and EGCG prevented erosive dentin wear.
Cardoso et al. (2020)	*In situ*	Bovine dentin	a. Proanthocyanidin mouthrinse (pH 7.0) b. Proanthocyanidin mouthrinse (pH 3.0)	No treatment	Surface loss (profilometry)	‐ Proanthocyanidin mouthrinse (pH 7.0): Decreased (*p* = 0.05). ‐ Proanthocyanidin mouthrinse (pH 3.0): No difference.
Jiang et al. (2020b)	*In vitro*	Human dentin	a. EGCG b. 75, 150, and 300 g/mL quercetin (Q75, Q150, and Q300)	Deionized water	1. Surface microhardness loss (Vickers hardness test) 2. Surface loss (profilometry) 3. Surface morphological changes (SEM)	1. All plant extracts: Decreased (*p* < 0.05).The Q300 group exhibited the least surface microhardness loss, which was significantly lower than those of the Q75 and Q150 groups.The Q75, Q150, and Q300 groups exhibited the least erosive dentin wear. 2. All plant extracts: Decreased (*p* < 0.05). 3. SEM images revealed that most of the dentinal tubules were exposed after acid challenges in the specimens in the control group, whereas the specimens treated with Q300 exhibited clear dentinal tubule occlusion.
Ozan et al. (2020)	*In situ*	Human dentin	a. Black tea b. Green tea	Water	1. Microhardness (Knoop hardness test) 2. Surface roughness (AFM)	1. Both plant extracts: Increased (*p* < 0.05). There was no significant difference between the green and black teas. 2. Both plant extracts: No difference (*p* < 0.05).AFM evaluations showed macromolecular deposits on the black tea group.Black tea showed similar surface characteristics as green tea.
Passos et al. (2018)	*In vitro* (human saliva)	Human dentin	a. EGCG b. Theaflavin gallate derivatives c. Commercial green tea d. Commercial black tea	Distilled water	Surface loss (profilometry)	All plant extracts: Decreased (*p* < 0.05).
De Moraes et al. (2016)	*In vitro*	Human dentin	Green tea	Distilled water	1. Surface hardness loss (Knoop hardness test) 2. Surface roughness (profilometry) 3. Surface loss (profilometry)	1. No difference. 2. Decreased (*p* < 0.001). 3. Decreased (*p* < 0.001).
Silveira et al. (2014)	*In vitro*	Bovine dentin	a. EGCG b. Saturated AA c. EGCG + saturated AA	No treatment	1. Surface loss (profilometry) 2. Surface morphological changes (SEM)	1. All plant extracts: Decreased (*p* < 0.0001).The addition of EGCG to saturated AA did not improve its effect against dentin surface loss.Solutions that contained saturated AA significantly reduced dentin loss compared to the EGCG solution. 2. The dentine samples from the control and EGCG groups showed wider dentinal tubules, while no differences were found for the other groups between the untreated and treated (exposed) dentin surfaces. For surfaces treated with saturated AA only, precipitation on intertubular dentine was observed.
Mirkarimi and Toomarian (2012)	*In vitro*	Human dentin	Green tea	No treatment	Surface morphological changes (SEM)	There was an improvement in eroded dentin appearance and deposits were present on the dentin surface.
Zhang et al. (2009)	*In vitro*	Bovine enamel	Galla chinensis	Distilled and deionized water	Surface morphological changes (SEM)	SEM image of galla chinensis extract‐treated enamel surface looked quite different from that of either the control group, some rod‐like deposits were disorderly distributed and formed many irregular prominences on the enamel surface.
Kato et al. (2009)	*In situ*	Bovine dentin	Green tea	Tap water (0.6–0.8 mg fluoride/L)	Surface loss (profilometry)	Decreased (*p* < 0.05).
Magalhães et al. (2009)	*In situ*	Bovine dentin	Green tea	Deionized water	Surface loss (profilometry)	Decreased (*p* < 0.05). The surface loss was significantly higher when the specimens were also abraded compared to the condition erosion only (*p* < 0.05).

Abbreviations: AA, anacardic acid; EGCG, epigallocatechin‐3‐gallate; SEM, scanning electron microscopy.

The active ingredients of the plant compounds assessed in the eligible studies is presented in Table S2.

#### 3.2.1 Preventive Effects

Several extracts demonstrated significant protective effects against erosive damage. Grape seed extract and green tea were among the most effective, with studies showing reduced surface loss and enhanced pellicle resilience. EGCG, a key compound in green tea, was repeatedly associated with decreased erosion rates.

#### 3.2.2 Therapeutic Outcomes

Studies investigating post‐erosion treatment showed more variable results. While some plant compounds provided a modest increase in surface microhardness or remineralization, the effects were typically less pronounced than observed in preventive applications.

### 3.3 Risk of Bias Within Studies

Of the 41 included studies, 11 presented a low risk of bias, 29 showed a medium risk of bias, and one was considered to have a high risk of bias according to the QUIN's parameters (Table 4).

**Table 4:** The results of the risk of bias assessment.

Parameter^a^														
Study	1	2	3	4	5	6	7	8	9	10	11	12	Final score	Risk
Schestakow et al. (2024)	2	0	2	2	2	0	0	2	2	0	2	2	66.6%	Medium
Martins et al. (2024)	2	1	2	2	2	0	2	2	2	0	2	2	79.16	Low
Baumann et al. (2023)	2	2	1	2	2	0	1	2	0	0	2	2	66.6%	Medium
Niemeyer et al. (2023a)	2	0	1	2	2	1	1	2	0	0	2	2	62.5%	Medium
Niemeyer et al. (2023b)	2	0	1	2	2	0	1	2	0	0	2	1	54.2%	Medium
Obeid et al. (2023)	2	0	1	2	2	0	0	2	0	0	2	2	54.2%	Medium
Rabelo et al. (2023)	2	2	2	2	2	0	2	2	0	0	2	2	75%	Low
Capalbo et al. (2022)	2	2	1	2	2	0	1	2	0	0	2	2	66.6%	Medium
Carvalho et al. (2022)	2	0	1	2	2	0	2	2	0	0	2	2	62.5%	Medium
Iftikhar et al. (2022)	2	2	1	2	2	0	1	2	0	0	2	2	66.6%	Medium
Kato et al. (2022)	2	0	1	2	2	0	1	2	0	0	2	2	58.3%	Medium
Li et al. (2022)	2	2	1	2	2	0	2	2	0	0	2	2	70.8%	Low
Schestakow et al. (2022)	2	1	1	1	2	0	0	2	0	0	2	2	54.2%	Medium
Hong et al. (2022)	2	0	2	2	2	1	2	2	0	1	2	2	75%	Low
Celik et al. (2021)	2	0	1	2	2	0	0	2	0	0	2	1	50.0%	Medium
DE Moraes et al. (2021)	2	2	2	2	2	0	2	2	0	2	2	2	83.3%	Low
Niemeyer et al. (2021)	2	0	1	2	2	0	0	2	0	0	2	2	54.2%	Medium
Rios et al. (2021)	2	2	2	2	2	0	2	2	0	0	2	2	75%	Low
Sarialioglu Gungor and Donmez (2021)	2	0	2	2	2	0	1	2	0	0	2	2	62.5%	Medium
Cardoso et al. (2020)	2	2	2	2	2	0	1	2	0	1	2	2	75%	Low
Jiang et al. (2020b)	2	0	2	2	2	0	2	2	0	0	2	2	66.6%	Medium
Ozan et al. (2020)	2	0	1	2	2	0	1	2	0	2	2	2	66.6%	Medium
Jiang et al. (2020a)	2	0	1	2	2	0	2	2	0	0	2	2	62.5%	Medium
Ionta et al. (2018)	2	1	2	2	2	1	1	2	0	2	2	2	79.2%	Low
Passos et al. (2018)	2	0	1	2	2	0	2	2	0	0	2	2	62.5%	Medium
Wang et al. (2018)	2	0	1	2	2	0	1	2	0	0	2	2	58.3%	Medium
Hertel et al. (2017)	2	0	1	2	2	0	0	1	0	0	2	2	50%	Medium
Ionta et al. (2017)	2	2	2	2	2	0	1	2	0	0	2	2	70.8%	Low
Sales‐Peres et al. (2016)	2	2	1	2	2	0	1	2	0	1	2	2	70.8%	Low
De Moraes et al. (2016)	2	0	1	2	2	0	2	2	0	1	2	2	66.6%	Medium
Hertel et al. (2016)	2	0	1	1	2	0	0	2	0	0	2	2	50%	Medium
Weber et al. (2015)	2	0	1	2	2	0	1	2	0	0	2	2	58.3%	Medium
Silveira et al. (2014)	2	0	2	2	2	0	1	2	0	0	2	2	62.5%	Medium
Hannig et al. (2012)	2	0	1	2	2	0	0	2	0	0	2	2	54.2%	Medium
Mirkarimi and Toomarian (2012)	2	0	1	0	1	0	0	2	0	0	2	2	41.6%	High
Guo et al. (2012)	2	0	1	2	2	0	2	2	0	1	2	2	66.6%	Medium
Souza et al. (2010)	2	0	1	2	2	0	1	2	0	0	2	2	58.3%	Medium
Zhang et al. (2009)	2	0	1	2	2	0	1	1	0	0	2	2	54.2%	Medium
Kato et al. (2009)	2	0	2	2	2	0	1	2	0	0	2	2	62.5%	Medium
Magalhães et al. (2009)	2	2	2	2	2	0	1	1	0	2	2	2	75%	Low
Wiegand et al. (2007)	2	0	1	2	2	0	2	1	0	0	2	2	58.3%	Medium

^a^ 1: Clearly stated aims/objectives, 2: detailed explanation of sample size calculation, 3: detailed explanation of sampling technique, 4: details of comparison group, 5: detailed explanation of methodology, 6: operator details, 7: randomization, 8: method of measurement of outcome, 9: outcome assessor details, 10: blinding, 11: statistical analysis, and 12: presentation of results.

### 3.4 Meta‐Analysis

Quantitative pooling was feasible only for dentin loss because enamel endpoints were too methodologically heterogeneous to combine and are summarized narratively in Tables 2 and 3, and in Section Conclusions, 4.

#### 3.4.1 Preventive Interventions on Dentin

**Figure 2:**
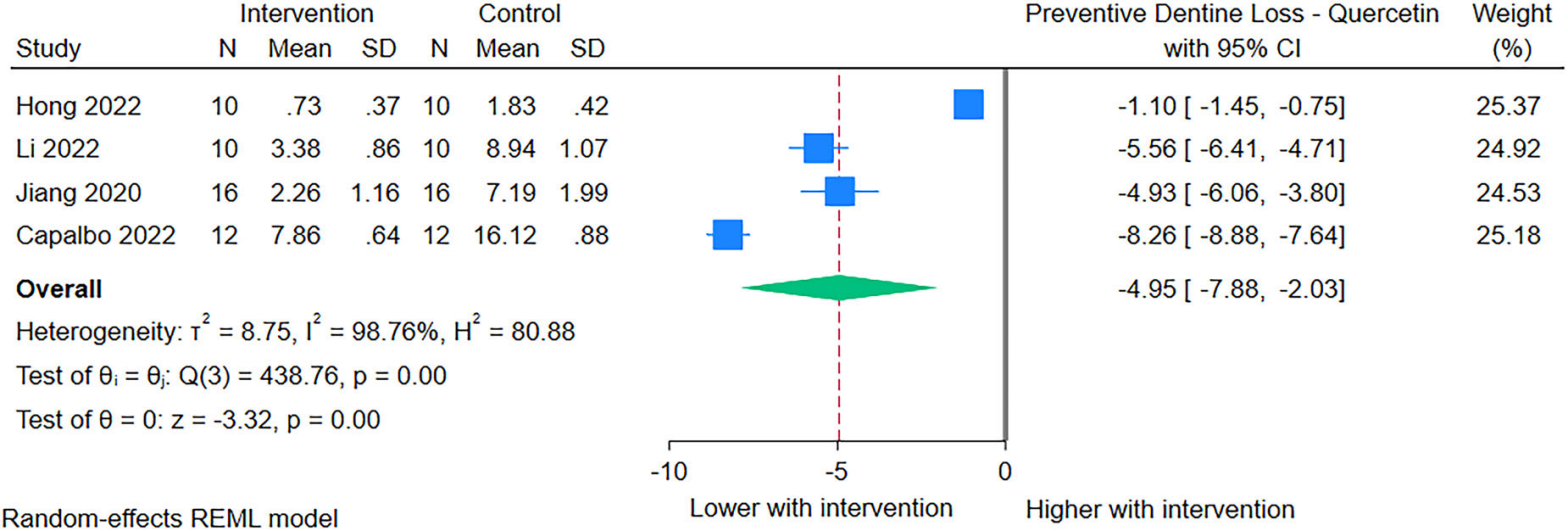
Results of the meta‐analysis for preventive effects of quercetin on dentin loss.

Based on four studies (Hong et al. 2022; Capalbo et al. 2022; Jiang et al. 2020a; Li et al. 2022) of 96 samples, the application of quercetin as a preventive measure before erosive interventions, significantly reduced dentin loss by 4.95 µm (95% CI: −7.88 to −2.03). These studies showed high heterogeneity (*I* ^2^ = 98%) (Figure 2).

#### 3.4.2 Therapeutic Interventions on Dentin

**Figure 3:**
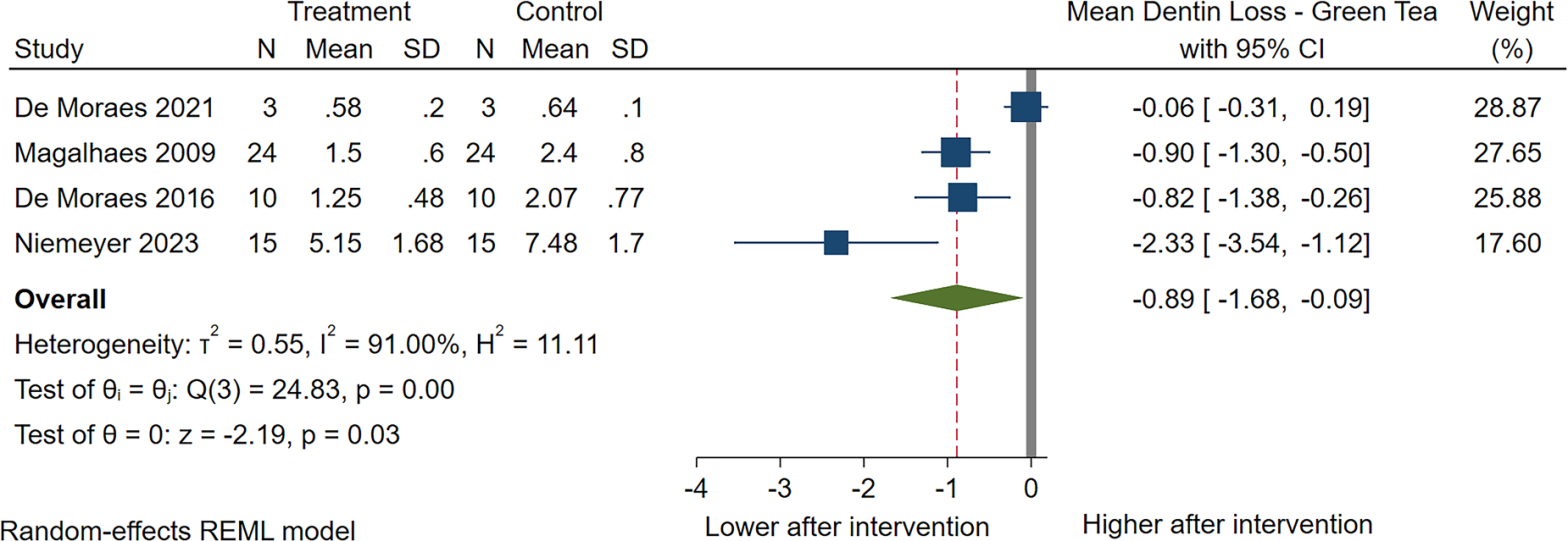
Results of the meta‐analysis for therapeutic effects of green tea on dentin loss.

The use of green tea was assessed in four studies (Niemeyer et al. 2023a; DE Moraes et al. 2021; De Moraes et al. 2016; Magalhães et al. 2009) for 104 total samples. Green tea significantly reduced dentin loss by 0.89 µm (95% CI: −1.68 to −0.09). However, these studies had a high degree of heterogeneity (*I* ^2^ = 91%) (Figure 3).

**Figure 4:**
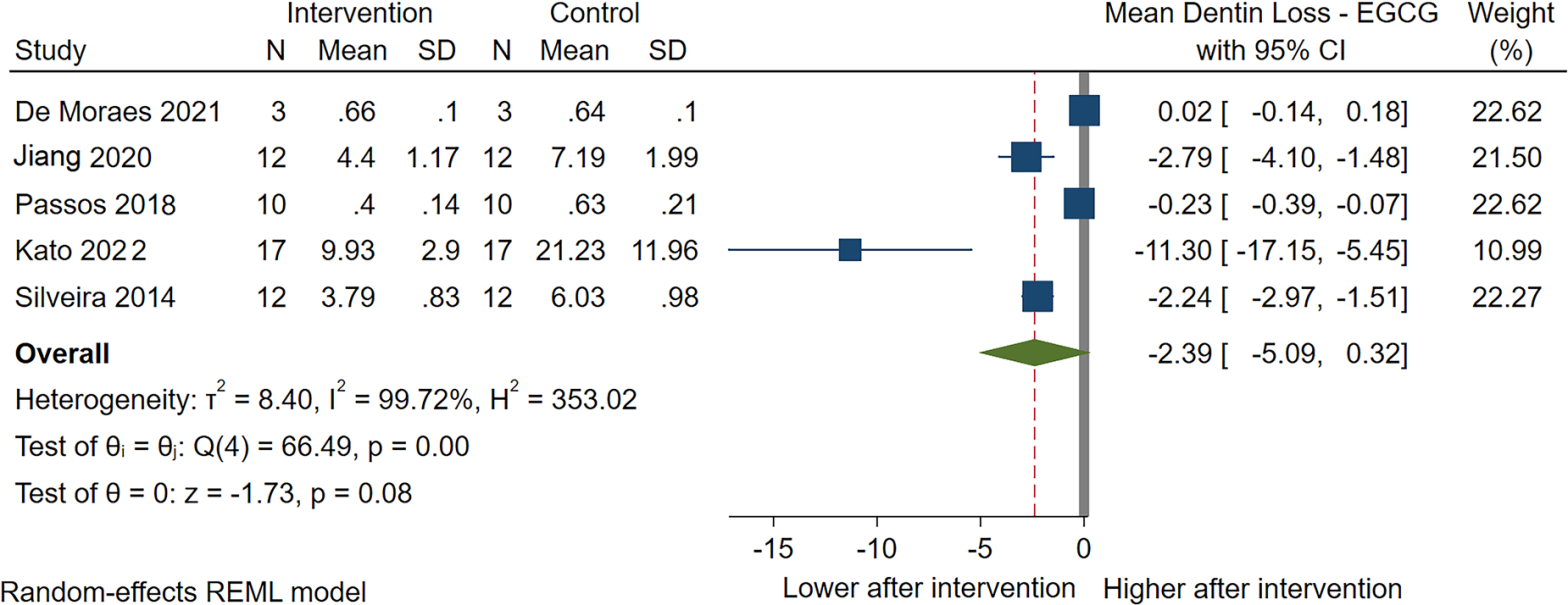
Results of the meta‐analysis for therapeutic effects of epigallocatechin‐3‐gallate on dentin loss.

EGCG was evaluated in five studies (Kato et al. 2022; DE Moraes et al. 2021; Jiang et al. 2020b; Passos et al. 2018; Silveira et al. 2014) on 108 total samples. Although EGCG reduced dentin loss, it was not statistically significantly (MD: −2.39, 95% CI: −5.09 to 0.32). These studies also exhibited high heterogeneity (*I* ^2^ = 99%) (Figure 4).

**Figure 5:**
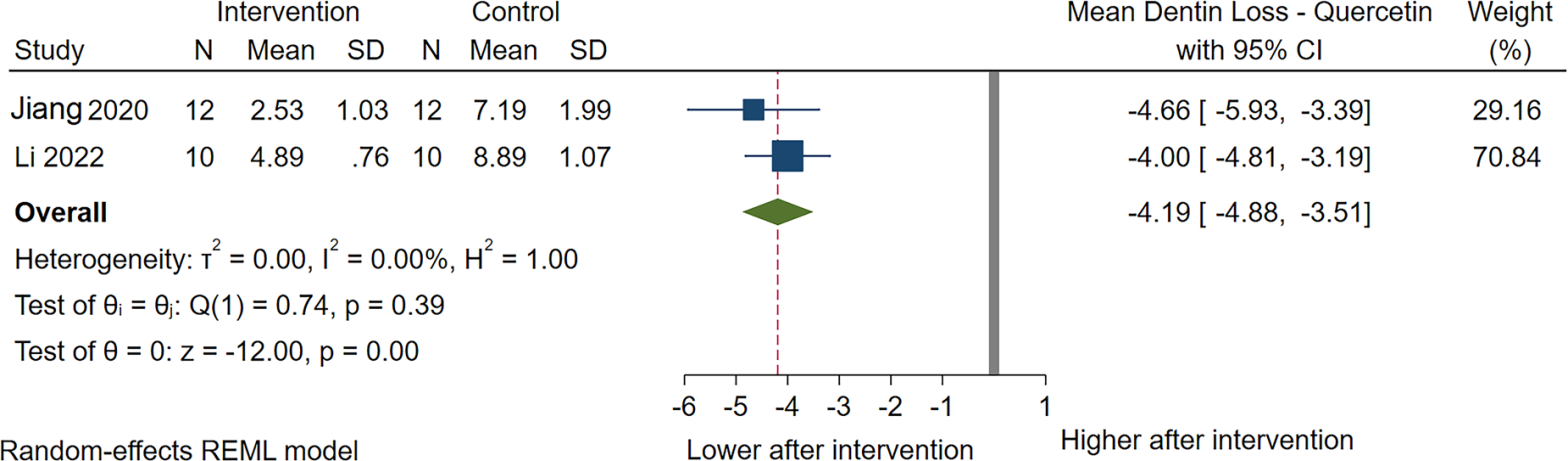
Results of the meta‐analysis for therapeutic effects of quercetin on dentin loss.

Based on two studies (Li et al. 2022; Jiang et al. 2020b) of 44 samples, the use of quercetin significantly reduced dentin loss by 4.19 µm (95% CI: −4.88 to −3.51). These studies showed low heterogeneity (*I* ^2^ = 0%) (Figure 5).

We performed a leave‐one‐out procedure because of the high statistical heterogeneity detected for two of the therapeutic interventions (green tea *I*² = 91%; EGCG *I*² = 99%) and the sole preventive intervention (quercetin *I*² = 98%) (Figures [Link], [Link]). For green tea, elimination of the individual studies shifted the pooled MD between −0.56 μm (exclusion of Niemeyer et al. 2023a, 2023b) and −1.16 μm (exclusion of de Moraes et al. 2021); the direction of effect was unchanged and remained significant in three of four iterations. For EGCG, sequential exclusions yielded pooled MDs from −1.22 to −3.31 μm, none of which reached statistical significance (*p* = 0.08–0.21). As for the quercetin (preventive protocol), pooled MDs ranged from −3.83 (Capalbo et al. 2022 omitted) to −6.29 µm (Hong et al. 2022 omitted); all iterations stayed significant and the overall estimate (−4.95 µm; *p* = 0.001) was essentially unaffected.

Furthermore, a risk‐of‐bias subgroup analysis (low vs. medium) did not materially change the conclusions or reduce heterogeneity. For quercetin (preventive), the pooled MD was −3.31 µm (95% CI: −7.68 to 1.06; *I*² = 98.9%) in low‐ROB studies and −6.63 µm (95% CI: −9.89 to −3.37; *I*² = 96.1%) in medium‐ROB studies, with no significant subgroup difference (Q_b = 1.42, *p* = 0.233) (Figure S4 **)**. For green tea (therapeutic), the low‐ROB subgroup was not significant (MD −0.47 µm; 95% CI: −1.29 to 0.36; *I*² = 91.7%), whereas the medium‐ROB subgroup remained borderline significant (MD −1.48 µm; 95% CI: −2.94 to −0.01; *I*² = 79.7%) and groups did not differ (Q_b = 1.39, *p* = 0.239) (Figure S5 **)**. Owing to the limited number of eligible studies, subgroup analyses were not feasible for the other comparisons. Taken together with our leave‐one‐out checks, these findings indicate that the direction of effect is consistent—and overall significant—for green‐tea extract and quercetin (though imprecise within the low‐ROB subsets), whereas evidence for EGCG remains inconclusive and highly variable across studies.

## 4 Discussion

This systematic review and meta‐analysis verified data from *in vitro* and *in situ* studies that assessed the preventive and therapeutic effects of plant‐derived compounds on enamel and dentin erosion.

Saliva is the most relevant biological factor that prevents dental erosion (Buzalaf et al. 2012), as it forms a salivary pellicle (SP), a nonbacterial organic layer on the enamel surface by absorbing proteins, peptides, lipids, and other salivary macromolecules, thereby reducing acid contact with teeth (Hannig and Hannig 2009, 2014). The outer layer of the acquired enamel pellicle (AEP) is easily removed after exposure to acid, whereas the basal layer may not be affected. The thickness and maturation time of the AEP influences its physical properties and capability for acid protection (Hannig and Hannig 2014). Plant‐derived compounds may prevent or treat dental erosion by improving mechanical properties and remineralization, or inhibit proteases and demineralization (Niemeyer et al. 2023a, 2021; Kato et al. 2022; Ozan et al. 2020). About 25% of the pellicle's dry weight consists of lipids (Slomiany et al. 1986); therefore, lipophilic components might modulate the composition of the pellicle and reduce erosion (Kensche et al. 2013). Plant oils may make the surface layer of the AEP rich in lipid micelles, which would make teeth more acid‐resistant (Das et al. 1976; Toro et al. 2000).

Polyphenols are a group of compounds present in plants that primarily affect changes to the pellicle. Polyphenol extracts could interact with the organic matrix of dentin and cross‐link collagen, and reduce its susceptibility to degradation (Sarialioglu Gungor and Donmez 2021; Mirkarimi and Toomarian 2012). In addition, they lead to thicker, stronger pellicles (Hiraishi et al. 2013). Matrix metalloproteinases (MMPs) belong to a group of zinc‐dependent proteins that may play a central role in the breakdown of the extracellular matrix. They exist in dentin and saliva, and MMP‐2, −8, and −9 are responsible for collagen breakdown in dentin (Pereira et al. 2016). After acid exposure, the dentin‐derived MMPs may be activated. The polyphenols are reported to have inhibitory properties against matrix MMP‐2 and −9 via hydrogen bonding and hydrophobic interactions. Therefore, MMPs are unable to disrupt the organic matrix of dentin under acidic conditions (Kato et al. 2010). In this review, we assessed different plant compounds and categorized them into fruit, leaf, flower, root, seed, tea, lipophilic, and other compounds.

### 4.1 Fruits (Blueberry, Cranberry, Pomegranate, and Açaí)

The blueberry extract had a better preventive effect against erosion compared to green tea or grape seed extract with pellicles. However, it did not show any therapeutic response due to decreased binding affinities to proteins in SP and collagen (Niemeyer et al. 2023a). Carvalho et al. (2022) reported that although blueberry alone had no therapeutic effects, blueberry and fluoride improved protection against enamel erosion. The therapeutic evaluation of cranberry showed a decrease in dentin erosion, which was comparable to green tea (Kato et al. 2022). Although pomegranates contain phenolic compounds, no preventive effect was observed on dentin erosion, and this might be attributed to the decreased pH (3.87) (Sarialioglu Gungor and Donmez 2021). Açaí extract did not protect against dentin erosion (Niemeyer et al. 2023a).

### 4.2 Leaves, Flowers, or Roots (Black Currant, Oregano, Hop, *Inula viscosa*, Moringa, Chinese Gall, Cloves, *Euclea natalensis*, Rosehips, and Ginger)

Rinses with *Ribes nigrum* (black current) and *Origanum vulgare* (oregano) before the acid challenge yielded thicker and more electron‐dense pellicles and might reduce dental erosion, especially for *Origanum vulgare*. *Origanum vulgare* compounds possibly aggregate salivary proteins and facilitate their adsorption to the enamel surface (Weber et al. 2015). Hop (or hops) extract increased the pellicle thickness. However, the polyphenol content of hop extract is low, thus it had less effect on the pellicle compared to fluoride or another polyphenolic compound (Schestakow et al. 2024). *Inula viscosa* tea altered the ultrastructure of the pellicle's basal layer. However, acid application decreased the density of the attached complexes and interrupted the previously treated basal layer (Hertel et al. 2016).

*Moringa oleifera* (Moringa) leaf extract has therapeutic effects because it promotes remineralization and inhibits demineralization of dentin (Obeid et al. 2023). The presence of phosphate and calcium in the leaves creates an alkaline environment and reduces the diameter of the dentinal tubules. In addition, the presence of flavonoids, amino acids, and oxalate may improve dentinal tubule occlusion (Epasinghe et al. 2016).

Galla chinensis (Chinese gall) includes polyphenolic compounds such as gallotannins and proanthocyanidins that prevent demineralization and remineralize early carious lesions. Moreover, gallic acid and methyl gallate form an enamel‐like remineralized surface layer with fluorine‐substituted hydroxyapatite crystals (Zhang et al. 2009).

The high amount of eugenol in clove tea prevents dentin erosion (Sarialioglu Gungor and Donmez 2021). Cloves increase osteocalcin in the media, and calcium granule formation may increase dentin hardness (Bakhori et al. 2019; Mendi et al. 2017). The roots of the Euclea species are rich in naphthoquinones and tannin, which may protect from demineralization (Kato et al. 2010). Sales‐Peres et al. (2016) reported that *Euclea natalensis* protected against dentin erosive wear but not enamel, which was possibly due to their different compositions. This plant extract can also show some therapeutic effects by contributing to MMP inhibition in dentin (Sales‐Peres et al. 2016). Pure rosehip tea did not show any preventive effects on enamel erosion (Sarialioglu Gungor and Donmez 2021). Niemeyer et al. (2021) reported that rosehip tea erodes enamel. Honey and chocolate combined with ginger can remineralize early enamel lesions but ginger alone is not protective (Celik et al. 2021).

### 4.3 Seeds (Grapefruit Seed Extract, Grape Seed, and Juca Seed Galactomannan)

The preventive effects of grapefruit seed extract against enamel erosion are possibly due to its flavanone content, mainly naringenin (Niemeyer et al. 2021; Carvalho et al. 2022). Baumann et al. (2023) and Sarialioglu Gungor and Donmez (2021) observed that grape seed alone had no protective effects on enamel or dentin. Niemeyer et al. (2023a, 2021) demonstrated that grape seed, when employed as a therapeutic agent, protected dentin and pellicle. However, it protected enamel, but not dentin when used as a preventive agent (Niemeyer et al. 2023a, 2021).

Caesalpinia ferrea, known as jucá, forms a protective layer on the enamel and dentin surfaces and has enzymatic inhibitory activities. According to Rabelo et al. (2023) the application of jucá after an erosive challenge did not prevent the progression of dentin erosion.

### 4.4 Black and Green Teas

Black and green teas both showed preventive and therapeutic effects on enamel and dentin erosion. The synergistic action of EGCG and theaflavin gallate derivatives, along with other bioactive compounds present in black and green teas, make them effective natural agents for remineralization and provide a protective effect on enamel and dentin against erosive challenges. Black tea contains fewer catechins than green tea, but more theaflavins and thearubigins that impart the reddish color to the tea (Gardner et al. 2007). Black tea has been shown to significantly reduce dentin erosion (Niemeyer et al. 2021; Schestakow et al. 2022; Ozan et al. 2020; Passos et al. 2018). Kato et al. found that the protective effect of green tea against dentin erosion was attributed to its phenolic content (EGCG), not fluoride (Kato et al. 2009). Although EGCG showed promising anti‐erosive effects in the included studies, our meta‐analysis did not demonstrate this effect to be statistically significant.

### 4.5 Lipophilic Components (Olive, Palm, Coconut, Safflower, Sunflower, and Peppermint Oils)

Lipid‐rich AEPs appear to be more resistant to acid challenges (Kensche et al. 2013). Research shows that olive oil prevents dental erosion differently compared to the control (Ionta et al. 2017; Wiegand et al. 2007; Buchalla et al. 2003). The types of olive oil, and its concentration, emulsion duration, tooth structure, demineralization process, and pellicle formation influence the mechanism of action of olive oil against tooth erosion. Wiegand et al. (2007) showed that a mouthrinse with 2% olive oil (Xerostom) decreased enamel demineralization, while pure olive oil (100%) did not reduce enamel or dentin erosion. Another study reported that olive oil did not have any protective effects on enamel (Ionta et al. 2017).

Pure palm oil also protects enamel against erosion (Rios et al. 2021; Ionta et al. 2018, 2017; Martins et al. 2024). The fatty acids in palm oil and AEPs are similar; therefore, they can easily combine to prevent tooth erosion (Reich et al. 2012). Martins et al. (2024) conducted research to evaluate the effects of palm oil on enamel protection against erosive and abrasive challenges. Tocotrienols in palm oil may allow it to enter and diffuse into the AEP basal layers, and increase its protective effect (Ahsan et al. 2015). Coconut, sunflower, and sunflower oils did not prevent enamel erosion. Studies show that the type and concentration of fatty acids in these oils may not protect the tooth and the pellicle. Sunflower has less tocotrienols than palm oil; and its effect is between olive, coconut, and safflower oils (Ionta et al. 2017). Peppermint tea did not show any preventive effect on enamel erosion which could be due to lower concentrations of flavones and flavanones compared to green and black teas (Niemeyer et al. 2021).

### 4.6 Others (Quercetin, Epigallocatechin Gallate, Theaflavin Gallate, Proanthocyanidin, Anacardic Acid [AA], Tannic Acid, Xylitol, and Propolis)

Quercetin is a polyphenolic flavonoid that has shown superior effects in protecting against dentin erosion compared to conventional treatments like sodium fluoride and chlorhexidine. This may be due to the dual function of quercetin as an MMP inhibitor and its crosslinking effect (Capalbo et al. 2022; Li et al. 2022; Jiang et al. 2020b). It is possible that the hydroxyl group of quercetin forms hydrogen bonds with the amide carbonyl or hydroxyl groups of collagen, which enhances the mechanical properties of the collagen matrix (Bedran‐Russo et al. 2011). Li et al. (2022) suggested that applying quercetin before erosive challenges is more effective in reducing dentin erosion compared to its therapeutic use, as it allows quercetin to penetrate and deposit on the dentin.

Black tea oxidation creates theaflavin gallate derivatives such as theaflavin‐3‐gallate and 3'‐gallate. The galloyl moiety of EGCG inhibits proteases, improves the mechanical properties of dentin, inhibits collagen degradation, and protects the SP against acid attacks (Vidal et al. 2014). The presence of its specific hydroxyl groups can also enhance its ability to bind with calcium ions, which is effective for future remineralization (Wang et al. 2018).

Proanthocyanidin by interacting with AEP proteins, can inactivate MMPs, reduce collagen degradation, and inhibit demineralization (Martins et al. 2024; Cardoso et al. 2020). The carboxylic groups of collagen fibrils form hydrogen bonds with the hydroxyl group on the proanthocyanidin aromatic rings. This bonding provides a structural advantage for proanthocyanidin compared to other polyphenols. It has been reported that a proanthocyanidin‐based mouth rinse from grape seed extract prevented dentin erosion better than chlorhexidine without chlorhexidine side effects (Cardoso et al. 2020).

Anacardic acid (AA) is the major phenolic component of cashew nutshell liquid (Omanakuttan et al. 2012). Silveria et al. observed that 1 min application of the AA solution after erosive challenge prevented the MMPs, and reduced the progression of dentin loss (Silveira et al. 2014).

Tannic acid has a preventive effect on dental erosion thanks to numerous functional groups that contain polyphenol (Schestakow et al. 2022, 2024; Hertel et al. 2017). Tannins can bind to proline‐rich proteins and histatins, which are found both in saliva and the pellicle (Bennick 2002).

Xylitol is a sugar alcohol that can form a complex with calcium ions on the dental surface, inhibit the translocation of dissolved calcium and phosphate, and penetrate into demineralized surfaces (Miake 2003). Enamel specimens pretreated with xylitol and subsequently subjected to erosive challenge had a decrease in surface loss (Souza et al. 2010).

Propolis did not show any preventive effects against enamel erosion. It is possible that other types of propolis extracts could increase the protective effects (Niemeyer et al. 2021).

Several key limitations should be addressed in this systematic study. The studies included *in vitro* or *in situ* research, which provides molecular insights but limits clinical usefulness. The results lack *in vivo* investigations, which limits their translational usefulness. The study design, including plant‐derived chemical type and concentration, application extract, tooth kinds (human and bovine), and measuring methods varied widely. Differences in plant‐based intervention pH levels provide inconsistent results. Inconsistencies made data pooling difficult and led to broad meta‐analyses confidence ranges. Clearly, more studies that use a comparable methodology are needed, as well as clinical trials to confirm these encouraging results.

## 5 Conclusion

Some natural plants have beneficial properties against tooth erosion. This review shows that *Ribes nigrum* and *Origanum vulgare* leaves, cloves and hop flowers, *Euclea natalensis* root, grapefruit seed and grape seed extracts have a preventive effect on tooth erosion. Cranberry fruit, grape seed, *Moringa oleifera*, and Galla chinensis leaves have a therapeutic effect on tooth erosion. Among the oils, only palm oil showed a preventive effect. Based on the meta‐analysis, quercetin has preventive and therapeutic effects, and green tea has therapeutic effects on dentin erosion. EGCG decreased dentin loss, but not significantly. It can be concluded that green tea (a rich source of catechin) and quercetin (the most popular flavonoid contained in vegetables and fruits) are good options instead of fluoride and other anti‐erosive substances.

## Author Contributions

Conceptualization: Mahtab Memarpour and Golnoush Farzinnia. Data curation: Neda Afzali Baghdadabadi, Golnoush Farzinnia, Mahya Agharokh, and Niloofar Mokhtari. Formal analysis: Mahtab Memarpour, Neda Afzali Baghdadabadi, and Erfan Bardideh. Investigation, writing – original draft, and writing – review and editing: Mahtab Memarpour, Neda Afzali Baghdadabadi, Golnoush Farzinnia, Mahya Agharokh, Niloofar Mokhtari, and Erfan Bardideh. Methodology: Mahtab Memarpour, Neda Afzali Baghdadabadi, and Golnoush Farzinnia. Project administration, supervision, validation, and visualization: Mahtab Memarpour. Resources: Mahtab Memarpour, Neda Afzali Baghdadabadi, and Golnoush Farzinnia. Software: Neda Afzali Baghdadabadi and Erfan Bardideh.

## Conflicts of Interest

The authors declare no conflicts of interest.

## Supporting information


**Supporting figure S1:** Sensitivity analysis for the therapeutic effects of EGCG on dentin loss.

**Supporting figure S2:** Sensitivity analysis for the therapeutic effects of green tea on dentin loss.

**Supporting figure S3:** Sensitivity analysis for the preventive effects of quercetin on dentin loss.

**Supporting figure S4:** Subgroup analysis based on risk of bias (low vs medium) for the preventive effects of quercetin on dentin loss.

**Supporting figure S5:** Subgroup analysis based on risk of bias (low vs medium) for the therapeutic effects of green tea on dentin loss.

**Table S1:** Studies excluded during the full‐text screening phase

**Table S2:** Active ingredients of the plant compounds.

## Data Availability

All data analyzed during this study are available from the corresponding author on reasonable request.
