# Supplementary figures and images for: Preventive and Therapeutic Effects of Plant‐Derived Compounds on Tooth Erosion: A Systematic Review and Meta‐Analysis of In Situ and In Vitro Studies
Source: Clin Exp Dent Res. 2025 Oct 28;11(6):e70235. doi: 10.1002/cre2.70235 (PMC12560117; doi:10.1002/cre2.70235)

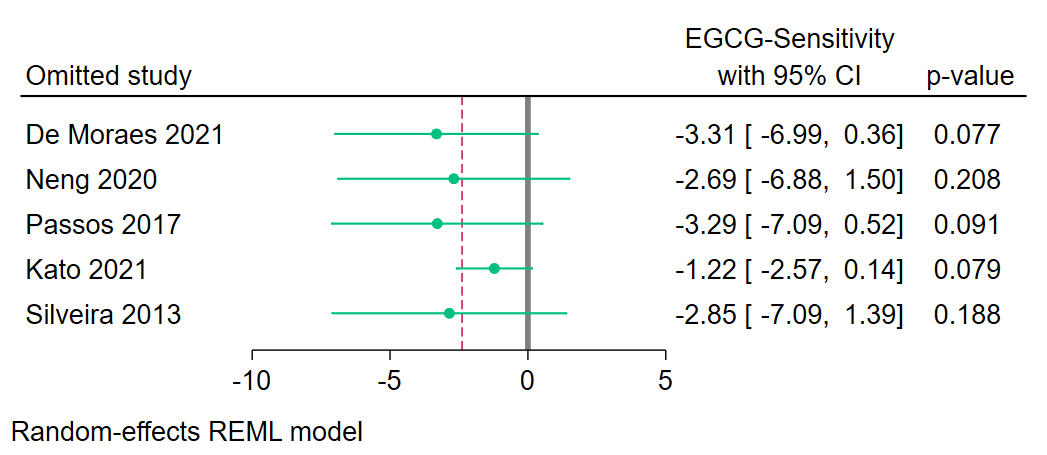

Supplement: Supplementary file 1 — Supporting figure S1: Sensitivity analysis for the therapeutic effects of EGCG on dentin loss. [file CRE2-11-e70235-s004.png]

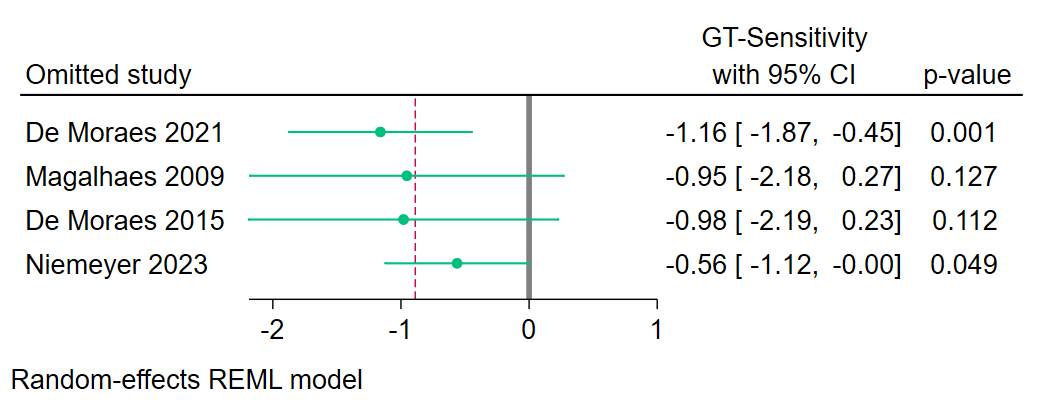

Supplement: Supplementary file 2 — Supporting figure S2: Sensitivity analysis for the therapeutic effects of green tea on dentin loss. [file CRE2-11-e70235-s001.png]

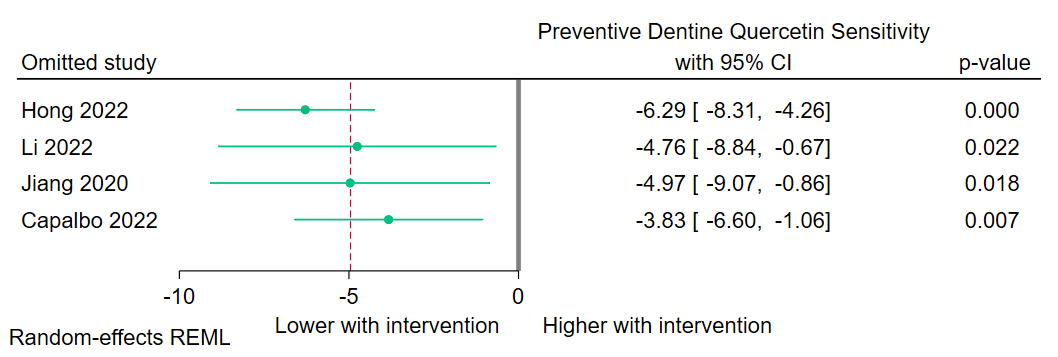

Supplement: Supplementary file 3 — Supporting figure S3: Sensitivity analysis for the preventive effects of quercetin on dentin loss. [file CRE2-11-e70235-s005.png]

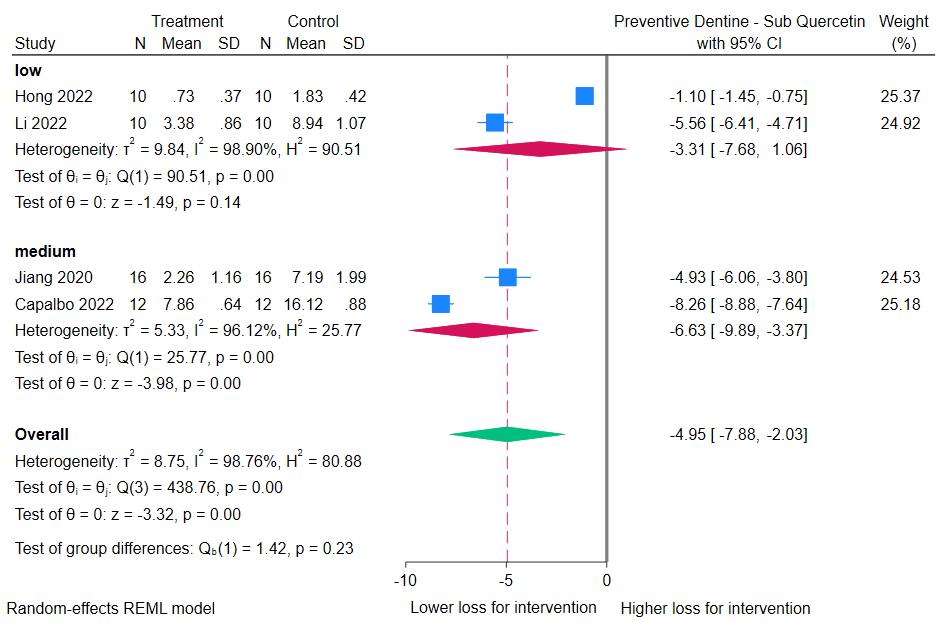

Supplement: Supplementary file 4 — Supporting figure S4: Subgroup analysis based on risk of bias (low vs medium) for the preventive effects of quercetin on dentin loss. [file CRE2-11-e70235-s003.png]

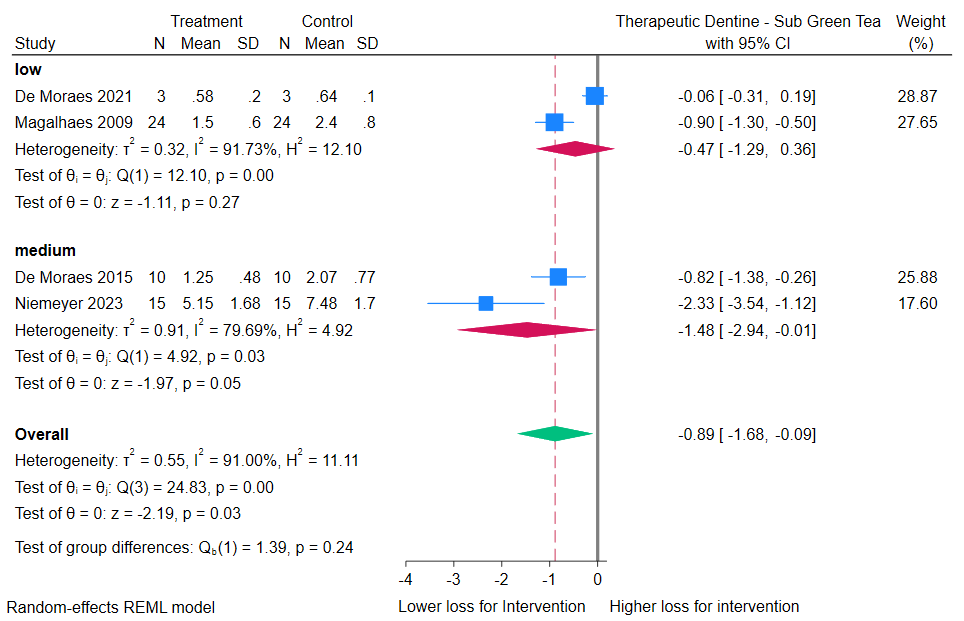

Supplement: Supplementary file 5 — Supporting figure S5: Subgroup analysis based on risk of bias (low vs medium) for the therapeutic effects of green tea on dentin loss. [file CRE2-11-e70235-s002.png]
