# Supplementary material for: Preventive and Therapeutic Effects of Plant‐Derived Compounds on Tooth Erosion: A Systematic Review and Meta‐Analysis of In Situ and In Vitro Studies
Source: Clin Exp Dent Res. 2025 Oct 28;11(6):e70235. doi: 10.1002/cre2.70235 (PMC12560117; doi:10.1002/cre2.70235)
Supplement: Supplementary file 6 — Table S1: Studies excluded during the full‐text screening phase [file CRE2-11-e70235-s007.docx]

**Table S1.** Studies excluded during the full-text screening phase

| **Reason of exclusion** | **Title** |
| --- | --- |
| **Negative control was not distilled/ deionized water or No control group** | 1. The roles of theaflavins in reducing dentin erosion |
|  | 1. Biomodification of eroded and abraded dentin with epigallocatechin-3-gallate (EGCG) |
|  | 1. The effect of red wine in modifying the salivary pellicle and modulating dental erosion kinetics |
|  | 1. [The effect of Salvadora Persica extract (miswak) and chlorahexidinegluconate on human dentin: A SEM study](https://https-www--scopus--com.daccess2.com/record/display.uri?eid=2-s2.0-3242809766&origin=resultslist&sort=plf-f&src=s&sid=a34657db0ea18943847d74cf661fad06&sot=a&sdt=cl&s=%28+%28+TITLE-ABS-KEY+%28+tea+%29+OR+TITLE-ABS-KEY+%28+%26apos%3B%26apos%3Bplant+AND+extracts%26apos%3B%26apos%3B+%29+OR+TITLE-ABS-KEY+%28+%26apos%3B%26apos%3Bplant+AND+oils%26apos%3B%26apos%3B+%29+OR+TITLE-ABS-KEY+%28+%26apos%3B%26apos%3Bplants%26apos%3B%26apos%3B+%29+OR+TITLE-ABS-KEY+%28+%26apos%3B%26apos%3Bpolyphenols%26apos%3B%26apos%3B+%29+OR+TITLE-ABS-KEY+%28+%26apos%3B%26apos%3Bepigallocatechin+AND+gallate%26apos%3B%26apos%3B+%29+OR+TITLE-ABS-KEY+%28+%26apos%3B%26apos%3Bcamellia+AND+sinensis%26apos%3B%26apos%3B+%29+%29+%29+AND+%28+%28+TITLE-ABS-KEY+%28+%26apos%3B%26apos%3Btooth+AND+erosion%26apos%3B%26apos%3B+%29+OR+TITLE-ABS-KEY+%28+%26apos%3B%26apos%3Benamel+AND+erosion%26apos%3B%26apos%3B+%29+OR+TITLE-ABS-KEY+%28+%26apos%3B%26apos%3Bdentin+AND+erosion%26apos%3B%26apos%3B+%29+OR+TITLE-ABS-KEY+%28+%26apos%3B%26apos%3Bdental+AND+erosion%26apos%3B%26apos%3B+%29+%29+%29&sl=667&sessionSearchId=a34657db0ea18943847d74cf661fad06&relpos=201) |
|  | 1. Supplementation of Energy Drinks with Green Tea Extract: Effect on In Vitro Abrasive/Erosive Dentin Wear |
|  | 1. Effect of Mouthrinses containing Olive Oil, Fluoride, and Their Combination on Enamel Erosion: An in vitro Study. |
|  | 1. [Green Tea Extract Reduces the Erosive Dentine Wear Caused by Energy Drinks In Vitro](https://https-www--scopus--com.daccess2.com/record/display.uri?eid=2-s2.0-85120557352&origin=resultslist&sort=plf-f&src=s&sid=a34657db0ea18943847d74cf661fad06&sot=a&sdt=cl&s=%28+%28+TITLE-ABS-KEY+%28+tea+%29+OR+TITLE-ABS-KEY+%28+%26apos%3B%26apos%3Bplant+AND+extracts%26apos%3B%26apos%3B+%29+OR+TITLE-ABS-KEY+%28+%26apos%3B%26apos%3Bplant+AND+oils%26apos%3B%26apos%3B+%29+OR+TITLE-ABS-KEY+%28+%26apos%3B%26apos%3Bplants%26apos%3B%26apos%3B+%29+OR+TITLE-ABS-KEY+%28+%26apos%3B%26apos%3Bpolyphenols%26apos%3B%26apos%3B+%29+OR+TITLE-ABS-KEY+%28+%26apos%3B%26apos%3Bepigallocatechin+AND+gallate%26apos%3B%26apos%3B+%29+OR+TITLE-ABS-KEY+%28+%26apos%3B%26apos%3Bcamellia+AND+sinensis%26apos%3B%26apos%3B+%29+%29+%29+AND+%28+%28+TITLE-ABS-KEY+%28+%26apos%3B%26apos%3Btooth+AND+erosion%26apos%3B%26apos%3B+%29+OR+TITLE-ABS-KEY+%28+%26apos%3B%26apos%3Benamel+AND+erosion%26apos%3B%26apos%3B+%29+OR+TITLE-ABS-KEY+%28+%26apos%3B%26apos%3Bdentin+AND+erosion%26apos%3B%26apos%3B+%29+OR+TITLE-ABS-KEY+%28+%26apos%3B%26apos%3Bdental+AND+erosion%26apos%3B%26apos%3B+%29+%29+%29&sl=667&sessionSearchId=a34657db0ea18943847d74cf661fad06&relpos=75) |
| **The plant extract combined with other ingredients** | 1. Effect of natural gel product on bovine dentin erosion in vitro |
|  | 1. [Effect of supplementation of soft drinks with green tea extract on their erosive potential against dentine](https://https-www--scopus--com.daccess2.com/record/display.uri?eid=2-s2.0-80052211573&origin=resultslist&sort=plf-f&src=s&sid=a34657db0ea18943847d74cf661fad06&sot=a&sdt=cl&s=%28+%28+TITLE-ABS-KEY+%28+tea+%29+OR+TITLE-ABS-KEY+%28+%26apos%3B%26apos%3Bplant+AND+extracts%26apos%3B%26apos%3B+%29+OR+TITLE-ABS-KEY+%28+%26apos%3B%26apos%3Bplant+AND+oils%26apos%3B%26apos%3B+%29+OR+TITLE-ABS-KEY+%28+%26apos%3B%26apos%3Bplants%26apos%3B%26apos%3B+%29+OR+TITLE-ABS-KEY+%28+%26apos%3B%26apos%3Bpolyphenols%26apos%3B%26apos%3B+%29+OR+TITLE-ABS-KEY+%28+%26apos%3B%26apos%3Bepigallocatechin+AND+gallate%26apos%3B%26apos%3B+%29+OR+TITLE-ABS-KEY+%28+%26apos%3B%26apos%3Bcamellia+AND+sinensis%26apos%3B%26apos%3B+%29+%29+%29+AND+%28+%28+TITLE-ABS-KEY+%28+%26apos%3B%26apos%3Btooth+AND+erosion%26apos%3B%26apos%3B+%29+OR+TITLE-ABS-KEY+%28+%26apos%3B%26apos%3Benamel+AND+erosion%26apos%3B%26apos%3B+%29+OR+TITLE-ABS-KEY+%28+%26apos%3B%26apos%3Bdentin+AND+erosion%26apos%3B%26apos%3B+%29+OR+TITLE-ABS-KEY+%28+%26apos%3B%26apos%3Bdental+AND+erosion%26apos%3B%26apos%3B+%29+%29+%29&sl=667&sessionSearchId=a34657db0ea18943847d74cf661fad06&relpos=164) |
|  | 1. [Hesperidin reduces dentin wear after erosion and erosion/abrasion cycling in vitro](https://https-www--scopus--com.daccess2.com/record/display.uri?eid=2-s2.0-85110529413&origin=resultslist&sort=plf-f&src=s&sid=a34657db0ea18943847d74cf661fad06&sot=a&sdt=cl&s=%28+%28+TITLE-ABS-KEY+%28+tea+%29+OR+TITLE-ABS-KEY+%28+%26apos%3B%26apos%3Bplant+AND+extracts%26apos%3B%26apos%3B+%29+OR+TITLE-ABS-KEY+%28+%26apos%3B%26apos%3Bplant+AND+oils%26apos%3B%26apos%3B+%29+OR+TITLE-ABS-KEY+%28+%26apos%3B%26apos%3Bplants%26apos%3B%26apos%3B+%29+OR+TITLE-ABS-KEY+%28+%26apos%3B%26apos%3Bpolyphenols%26apos%3B%26apos%3B+%29+OR+TITLE-ABS-KEY+%28+%26apos%3B%26apos%3Bepigallocatechin+AND+gallate%26apos%3B%26apos%3B+%29+OR+TITLE-ABS-KEY+%28+%26apos%3B%26apos%3Bcamellia+AND+sinensis%26apos%3B%26apos%3B+%29+%29+%29+AND+%28+%28+TITLE-ABS-KEY+%28+%26apos%3B%26apos%3Btooth+AND+erosion%26apos%3B%26apos%3B+%29+OR+TITLE-ABS-KEY+%28+%26apos%3B%26apos%3Benamel+AND+erosion%26apos%3B%26apos%3B+%29+OR+TITLE-ABS-KEY+%28+%26apos%3B%26apos%3Bdentin+AND+erosion%26apos%3B%26apos%3B+%29+OR+TITLE-ABS-KEY+%28+%26apos%3B%26apos%3Bdental+AND+erosion%26apos%3B%26apos%3B+%29+%29+%29&sl=667&sessionSearchId=a34657db0ea18943847d74cf661fad06&relpos=68) |
|  | 1. [Development of Epigallocatechin-3-gallate-Encapsulated Nanohydroxyapatite/Mesoporous Silica for Therapeutic Management of Dentin Surface](https://https-www--scopus--com.daccess2.com/record/display.uri?eid=2-s2.0-85027279985&origin=resultslist&sort=plf-f&src=s&sid=a34657db0ea18943847d74cf661fad06&sot=a&sdt=cl&s=%28+%28+TITLE-ABS-KEY+%28+tea+%29+OR+TITLE-ABS-KEY+%28+%26apos%3B%26apos%3Bplant+AND+extracts%26apos%3B%26apos%3B+%29+OR+TITLE-ABS-KEY+%28+%26apos%3B%26apos%3Bplant+AND+oils%26apos%3B%26apos%3B+%29+OR+TITLE-ABS-KEY+%28+%26apos%3B%26apos%3Bplants%26apos%3B%26apos%3B+%29+OR+TITLE-ABS-KEY+%28+%26apos%3B%26apos%3Bpolyphenols%26apos%3B%26apos%3B+%29+OR+TITLE-ABS-KEY+%28+%26apos%3B%26apos%3Bepigallocatechin+AND+gallate%26apos%3B%26apos%3B+%29+OR+TITLE-ABS-KEY+%28+%26apos%3B%26apos%3Bcamellia+AND+sinensis%26apos%3B%26apos%3B+%29+%29+%29+AND+%28+%28+TITLE-ABS-KEY+%28+%26apos%3B%26apos%3Btooth+AND+erosion%26apos%3B%26apos%3B+%29+OR+TITLE-ABS-KEY+%28+%26apos%3B%26apos%3Benamel+AND+erosion%26apos%3B%26apos%3B+%29+OR+TITLE-ABS-KEY+%28+%26apos%3B%26apos%3Bdentin+AND+erosion%26apos%3B%26apos%3B+%29+OR+TITLE-ABS-KEY+%28+%26apos%3B%26apos%3Bdental+AND+erosion%26apos%3B%26apos%3B+%29+%29+%29&sl=667&sessionSearchId=a34657db0ea18943847d74cf661fad06&relpos=110) |
|  | 1. [Effect of beverages on bovine dental enamel subjected to erosive challenge with hydrochloric acid](https://https-www--scopus--com.daccess2.com/record/display.uri?eid=2-s2.0-84870931696&origin=resultslist&sort=plf-f&src=s&sid=a34657db0ea18943847d74cf661fad06&sot=a&sdt=cl&s=%28+%28+TITLE-ABS-KEY+%28+tea+%29+OR+TITLE-ABS-KEY+%28+%26apos%3B%26apos%3Bplant+AND+extracts%26apos%3B%26apos%3B+%29+OR+TITLE-ABS-KEY+%28+%26apos%3B%26apos%3Bplant+AND+oils%26apos%3B%26apos%3B+%29+OR+TITLE-ABS-KEY+%28+%26apos%3B%26apos%3Bplants%26apos%3B%26apos%3B+%29+OR+TITLE-ABS-KEY+%28+%26apos%3B%26apos%3Bpolyphenols%26apos%3B%26apos%3B+%29+OR+TITLE-ABS-KEY+%28+%26apos%3B%26apos%3Bepigallocatechin+AND+gallate%26apos%3B%26apos%3B+%29+OR+TITLE-ABS-KEY+%28+%26apos%3B%26apos%3Bcamellia+AND+sinensis%26apos%3B%26apos%3B+%29+%29+%29+AND+%28+%28+TITLE-ABS-KEY+%28+%26apos%3B%26apos%3Btooth+AND+erosion%26apos%3B%26apos%3B+%29+OR+TITLE-ABS-KEY+%28+%26apos%3B%26apos%3Benamel+AND+erosion%26apos%3B%26apos%3B+%29+OR+TITLE-ABS-KEY+%28+%26apos%3B%26apos%3Bdentin+AND+erosion%26apos%3B%26apos%3B+%29+OR+TITLE-ABS-KEY+%28+%26apos%3B%26apos%3Bdental+AND+erosion%26apos%3B%26apos%3B+%29+%29+%29&sl=667&sessionSearchId=a34657db0ea18943847d74cf661fad06&relpos=162) |
|  | 1. Effect of pellicle modification with polyphenol-rich solutions on enamel  erosion and abrasion |
|  | 1. Pilot study of the effect of green tea extractive epigallocatechin-3-gallate  on degradation of collagen in dental erosion |
|  | 1. Evaluation of Proanthocyanidin-based dentifrices on dentin-wear after erosion and dental |
| **It did not contain any plant derivatives** | 1. [A New Sugarcane Cystatin Strongly Binds to Dental Enamel and Reduces Erosion](https://https-www--scopus--com.daccess2.com/record/display.uri?eid=2-s2.0-85025658426&origin=resultslist&sort=plf-f&src=s&sid=a34657db0ea18943847d74cf661fad06&sot=a&sdt=cl&s=%28+%28+TITLE-ABS-KEY+%28+tea+%29+OR+TITLE-ABS-KEY+%28+%26apos%3B%26apos%3Bplant+AND+extracts%26apos%3B%26apos%3B+%29+OR+TITLE-ABS-KEY+%28+%26apos%3B%26apos%3Bplant+AND+oils%26apos%3B%26apos%3B+%29+OR+TITLE-ABS-KEY+%28+%26apos%3B%26apos%3Bplants%26apos%3B%26apos%3B+%29+OR+TITLE-ABS-KEY+%28+%26apos%3B%26apos%3Bpolyphenols%26apos%3B%26apos%3B+%29+OR+TITLE-ABS-KEY+%28+%26apos%3B%26apos%3Bepigallocatechin+AND+gallate%26apos%3B%26apos%3B+%29+OR+TITLE-ABS-KEY+%28+%26apos%3B%26apos%3Bcamellia+AND+sinensis%26apos%3B%26apos%3B+%29+%29+%29+AND+%28+%28+TITLE-ABS-KEY+%28+%26apos%3B%26apos%3Btooth+AND+erosion%26apos%3B%26apos%3B+%29+OR+TITLE-ABS-KEY+%28+%26apos%3B%26apos%3Benamel+AND+erosion%26apos%3B%26apos%3B+%29+OR+TITLE-ABS-KEY+%28+%26apos%3B%26apos%3Bdentin+AND+erosion%26apos%3B%26apos%3B+%29+OR+TITLE-ABS-KEY+%28+%26apos%3B%26apos%3Bdental+AND+erosion%26apos%3B%26apos%3B+%29+%29+%29&sl=667&sessionSearchId=a34657db0ea18943847d74cf661fad06&relpos=112) |
|  | 1. Remineralization effect of three different agents on initial caries and erosive lesions: a micro-computed tomography and scanning electron microscopy analysis |
|  | The erosive potential of soft drinks on enamel surface substrate: an in vitro scanning electron microscopy investigation |
| **Different methodology or test** | 1. Antierosive profile of an experimental solution based on antioxidants from Passiflora edulis on initial dentin erosion lesions |
|  | Proanthocyanidin protects the enamel against initial erosive challenge when applied over acquired pellicle |
|  | Comparative evaluation of grape seed and cranberry extracts in preventing enamel erosion: An optical emission spectrometric analysis |
|  | 1. Pellicle modification with natural bioproducts: Influence on tooth color  under erosive conditions |
|  | 1. [Souakine mouth rinse solution protects deciduous enamel from simulated erosion in vitro](https://https-www--scopus--com.daccess2.com/record/display.uri?eid=2-s2.0-84928590923&origin=resultslist&sort=plf-f&src=s&sid=a34657db0ea18943847d74cf661fad06&sot=a&sdt=cl&s=%28+%28+TITLE-ABS-KEY+%28+tea+%29+OR+TITLE-ABS-KEY+%28+%26apos%3B%26apos%3Bplant+AND+extracts%26apos%3B%26apos%3B+%29+OR+TITLE-ABS-KEY+%28+%26apos%3B%26apos%3Bplant+AND+oils%26apos%3B%26apos%3B+%29+OR+TITLE-ABS-KEY+%28+%26apos%3B%26apos%3Bplants%26apos%3B%26apos%3B+%29+OR+TITLE-ABS-KEY+%28+%26apos%3B%26apos%3Bpolyphenols%26apos%3B%26apos%3B+%29+OR+TITLE-ABS-KEY+%28+%26apos%3B%26apos%3Bepigallocatechin+AND+gallate%26apos%3B%26apos%3B+%29+OR+TITLE-ABS-KEY+%28+%26apos%3B%26apos%3Bcamellia+AND+sinensis%26apos%3B%26apos%3B+%29+%29+%29+AND+%28+%28+TITLE-ABS-KEY+%28+%26apos%3B%26apos%3Btooth+AND+erosion%26apos%3B%26apos%3B+%29+OR+TITLE-ABS-KEY+%28+%26apos%3B%26apos%3Benamel+AND+erosion%26apos%3B%26apos%3B+%29+OR+TITLE-ABS-KEY+%28+%26apos%3B%26apos%3Bdentin+AND+erosion%26apos%3B%26apos%3B+%29+OR+TITLE-ABS-KEY+%28+%26apos%3B%26apos%3Bdental+AND+erosion%26apos%3B%26apos%3B+%29+%29+%29&sl=667&sessionSearchId=a34657db0ea18943847d74cf661fad06&relpos=138) |
|  | 1. [Erosive potential of industrialized teas: An in vitro study](https://https-www--scopus--com.daccess2.com/record/display.uri?eid=2-s2.0-85027104152&origin=resultslist&sort=plf-f&src=s&sid=a34657db0ea18943847d74cf661fad06&sot=a&sdt=cl&s=%28+%28+TITLE-ABS-KEY+%28+tea+%29+OR+TITLE-ABS-KEY+%28+%26apos%3B%26apos%3Bplant+AND+extracts%26apos%3B%26apos%3B+%29+OR+TITLE-ABS-KEY+%28+%26apos%3B%26apos%3Bplant+AND+oils%26apos%3B%26apos%3B+%29+OR+TITLE-ABS-KEY+%28+%26apos%3B%26apos%3Bplants%26apos%3B%26apos%3B+%29+OR+TITLE-ABS-KEY+%28+%26apos%3B%26apos%3Bpolyphenols%26apos%3B%26apos%3B+%29+OR+TITLE-ABS-KEY+%28+%26apos%3B%26apos%3Bepigallocatechin+AND+gallate%26apos%3B%26apos%3B+%29+OR+TITLE-ABS-KEY+%28+%26apos%3B%26apos%3Bcamellia+AND+sinensis%26apos%3B%26apos%3B+%29+%29+%29+AND+%28+%28+TITLE-ABS-KEY+%28+%26apos%3B%26apos%3Btooth+AND+erosion%26apos%3B%26apos%3B+%29+OR+TITLE-ABS-KEY+%28+%26apos%3B%26apos%3Benamel+AND+erosion%26apos%3B%26apos%3B+%29+OR+TITLE-ABS-KEY+%28+%26apos%3B%26apos%3Bdentin+AND+erosion%26apos%3B%26apos%3B+%29+OR+TITLE-ABS-KEY+%28+%26apos%3B%26apos%3Bdental+AND+erosion%26apos%3B%26apos%3B+%29+%29+%29&sl=667&sessionSearchId=a34657db0ea18943847d74cf661fad06&relpos=117) |
|  | 1. Erosive potential of vitamin waters, herbal drinks, carbonated soft drinks, and fruit juices on human teeth: An in vitro investigation |
|  | 1. Oral astringent stimuli alter the enamel pellicle's ultrastructure as  revealed by electron microscopy |
| **RCT** | 1. [Effect of different concentrations and application times of proanthocyanidin gels on dentin erosion.](https://pubmed.ncbi.nlm.nih.gov/29178771/) |

**References**

1. Guo J, Yang M, Hu M. The roles of theaflavins in reducing dentin erosion. *Sci Rep*. 2023;13(1):9413.
2. Abreu BD, Scatolin RS, Corona SAM, Curylofo Zotti FA. Biomodification of eroded and abraded dentin with epigallocatechin-3-gallate (EGCG). *J Mech Behav Biomed Mater*. 2023;147:106158.
3. Carvalho TS, Pham KN, Niemeyer SH, Baumann T. The effect of red wine in modifying the salivary pellicle and modulating dental erosion kinetics. *Eur J Oral Sci*. 2021;129(1):e12749.
4. Almas K. The effect of Salvadora persica extract (miswak) and chlorhexidine gluconate on human dentin: a SEM study. *J Contemp Dent Pract*. 2002;3(3):27-35.
5. Blatter N, Hamza B, Attin T, Wegehaupt FJ. Supplementation of Energy Drinks with Green Tea Extract: Effect on In Vitro Abrasive/Erosive Dentin Wear. *Oral Health Prev Dent*. 2023;21:391-396.
6. Vivek HP, Prashant GM, Geetha S, Chandramohan S, Imranulla M, Srinidhi PB. Effect of Mouthrinses containing Olive Oil, Fluoride, and Their Combination on Enamel Erosion: An in vitro Study. *J Contemp Dent Pract*. 2018;19(2):130-136.
7. Hamza B, Rojas SAP, Körner P, Attin T, Wegehaupt FJ. Green Tea Extract Reduces the Erosive Dentine Wear Caused by Energy Drinks In Vitro. *Oral Health Prev Dent*. 2021;19:573-578.
8. Sales-Peres Ade C, Marsicano JA, Garcia RP, Forim MR, Silva MF, Sales-Peres SH. Effect of natural gel product on bovine dentin erosion in vitro. *J Appl Oral Sci*. 2013;21(6):597-600.
9. Barbosa CS, Kato MT, Buzalaf MA. Effect of supplementation of soft drinks with green tea extract on their erosive potential against dentine. *Aust Dent J*. 2011;56(3):317-321.
10. Leal IC, Rabelo CS, Viana ÍEL, Scaramucci T, Santiago SL, Passos VF. Hesperidin reduces dentin wear after erosion and erosion/abrasion cycling in vitro. *Arch Oral Biol*. 2021;129:105208.
11. Yu J, Yang H, Li K, Ren H, Lei J, Huang C. Development of Epigallocatechin-3-gallate-Encapsulated Nanohydroxyapatite/Mesoporous Silica for Therapeutic Management of Dentin Surface. *ACS Appl Mater Interfaces*. 2017;9(31):25796-25807.
12. Amoras DR, Corona SA, Rodrigues AL Jr, Serra MC. Effect of beverages on bovine dental enamel subjected to erosive challenge with hydrochloric acid. *Braz Dent J*. 2012;23(4):367-372.
13. Mailart MC, Berisha I, Reinales ASA, et al. Effect of pellicle modification with polyphenol-rich solutions on enamel erosion and abrasion. *Braz Oral Res*. 2025;39:e024.
14. Chen H, Huang B. *Hua Xi Kou Qiang Yi Xue Za Zhi*. 2012;30(5):549-551.
15. Bueno TL, da Silva TA, Rizzante FA, Magalhães AC, Rios D, Honório HM. Evaluation of Proanthocyanidin-based dentifrices on dentin-wear after erosion and dental abrasion - *In situ* study. *J Clin Exp Dent*. 2022;14(4):e366-e370.
16. Santiago AC, Khan ZN, Miguel MC, et al. A New Sugarcane Cystatin Strongly Binds to Dental Enamel and Reduces Erosion. *J Dent Res*. 2017;96(9):1051-1057.
17. Akküç S, Duruk G, Keleş A. Remineralization effect of three different agents on initial caries and erosive lesions: a micro-computed tomography and scanning electron microscopy analysis. *BMC Oral Health*. 2023;23(1):106.
18. Owens BM, Kitchens M. The erosive potential of soft drinks on enamel surface substrate: an in vitro scanning electron microscopy investigation. *J Contemp Dent Pract*. 2007;8(7):11-20.
19. Marvão RM, Louzeiro TL, Alves MC, Oliveira KF, Goto GT, Fernandes GC, Barros AP, Kuga MC, Alencar CD. Antierosive profile of an experimental solution based on antioxidants from Passiflora edulis on initial dentin erosion lesions. Revista de Odontologia da UNESP. 2024 Aug 19;53:e20240005.
20. Boteon AP, Dallavilla GG, Cardoso F, Wang L, Rios D, Honório HM. Proanthocyanidin protects the enamel against initial erosive challenge when applied over acquired pellicle. *Am J Dent*. 2020;33(5):239-242.
21. Nandakumar M, Nasim I. Comparative evaluation of grape seed and cranberry extracts in preventing enamel erosion: An optical emission spectrometric analysis. *J Conserv Dent*. 2018;21(5):516-520.
22. Mailart MC, Berli PC, Borges AB, Yilmaz B, Baumann T, Carvalho TS. Pellicle modification with natural bioproducts: Influence on tooth color under erosive conditions. *Eur J Oral Sci*. 2022;130(5):e12886.
23. M'Barek Z, Zouari Y, Moalla N, Zaier E, Ghoul-Mazgar S. Souakine mouth rinse solution protects deciduous enamel from simulated erosion in vitro. *Eur J Paediatr Dent*. 2014;15(4):407-411.
24. de Oliveira AF, Sampaio FC, Meira IA, Bezerra MG, Fernandes NL, Paiva VM. Erosive potential of industrialized teas: An in vitro study. Pesquisa brasileira em odontopediatria e clínica integrada. 2017;17(1):1-7.
25. Surarit R, Jiradethprapai K, Lertsatira K, Chanthongthiti J, Teanchai C, Horsophonphong S. Erosive potential of vitamin waters, herbal drinks, carbonated soft drinks, and fruit juices on human teeth: An in vitro investigation. *J Dent Res Dent Clin Dent Prospects*. 2023;17(3):129-135.
26. Rehage M, Delius J, Hofmann T, Hannig M. Oral astringent stimuli alter the enamel pellicle's ultrastructure as revealed by electron microscopy. *J Dent*. 2017;63:21-29.
27. Boteon AP, Prakki A, Rabelo Buzalaf MA, Rios D, Honorio HM. Effect of different concentrations and application times of proanthocyanidin gels on dentin erosion. *Am J Dent*. 2017;30(2):96-100.
