# Supplementary material for: Preventive and Therapeutic Effects of Plant‐Derived Compounds on Tooth Erosion: A Systematic Review and Meta‐Analysis of In Situ and In Vitro Studies
Source: Clin Exp Dent Res. 2025 Oct 28;11(6):e70235. doi: 10.1002/cre2.70235 (PMC12560117; doi:10.1002/cre2.70235)
Supplement: Supplementary file 7 — Table S2: Active ingredients of the plant compounds. [file CRE2-11-e70235-s006.docx]

**Table S2.** **Active ingredients of the plant compounds.**

| **Part of plant extract** | **Main active substances** | **Preventive**  **effect** | **Therapeutic**  **effect** |
| --- | --- | --- | --- |
| **Fruit** |  |  |  |
| Vaccinium sect. Cyanococcus (Blueberry) [[1-4](#_ENREF_1)] | Anthocyanins (malvidin-3-glucoside/galactoside), phenolic acids, procyanidins, derivates of stilbenes, flavonols (kaempferol, quercetin, myricetin, fisetin) | In the presence of pellicle, blueberry showed protection | **-** |
| Vaccinium subg. Oxycoccus (Cranberry) [[5-8](#_ENREF_5)] | Proanthocyanidins (polymeric proanthocyanidin), anthocyanins, catechins, phenolic acids | **-** | **+** |
| Punica granatum (Pomegranates)[[9](#_ENREF_9), [10](#_ENREF_10)] | Anthocyanins (delphinidin, cyanidin, pelargonidin, glycosides), flavonoids (prunin, catechin, chrysin, apigenin, biochanin, luteolin, quercetin, kaempferol, taxifolin), tannins, proanthocyanidins, terpenes and terpenoids, lignans, xanthonoids, phenolic acids | **-** | NS* |
| Euterpe oleracea (Açaí) [[1](#_ENREF_1), [11](#_ENREF_11)] | Anthocyanins (cyanidin 3-glucoside/rutinoside), flavonoids, proanthocyanidins, phenolic acids (ferulic, vanillic and gallic acids) | NS | **-** |
| **Leaf, flower or root** |  |  |  |
| Origanum vulgare leaves )Oregano( [[12](#_ENREF_12)] | Anthocyanins, tannins, chlorogenic, protocatechuic, rosmarinic, neochlorogenic acid, quercetin glucosides, kaempferol and luteolin derivatives | **+** | NS |
| Ribes nigrum leaves (Black Currant) [[12](#_ENREF_12), [13](#_ENREF_13)] | Flavonoids (camphor oil, quercetin, myricetin, isorhamnetin), proanthocyanidins (catechin, epicatechin), anthocyanins (delphinidin, cyanidin), lignoids, phenolic acids | **+** | NS |
| Moringa oleifera leaves (Moringa) [[14-16](#_ENREF_14)] | Flavonoids (apigenin, quercetin, luteolin, myricetin, kaempferol), lignans (secoisolari-ciresinol, isolariciresinol, medioresinol, epipinoresinol glycosides), phenolcarboxylic acids and their derivatives, phenolic acid, proanthocyanidins, tannins, saponins | NS | **+** |
| Dittrichia viscosa (Inula viscosa  leaves or False yellowhead) [[17-19](#_ENREF_17)] | Flavonoids (nepetin, 3-O-methylquercetin, 3, 3′-di-O-methylquercetin, hispidulin, flavanone Sakuranetin, naringenin, luteolin, kaempferol), phenolic acids (caffeic, p-cinnamic, ferulic, chlorogenic and gallic acids) | **-** | NS |
| Galla chinensis  leaves (Chinese gall)[[20-22](#_ENREF_20)] | Rich in tannins (hydrolysable tannin comprise glucogallin, gallotannins, ella-gitannins), methyl gallate, phenolic acid (gallic, proto-catechuic, 2-hydroxy-6-pentadecyl benzoic and 4-hydroxy-3-methoxybenzoic acids) | **+** | **+** |
| Rosa canina L. flower (Rosehip) [[7](#_ENREF_7), [9](#_ENREF_9), [23](#_ENREF_23)] | Flavonols, flavanols, flavone, flavanones, tannins, anthocyanin, phenolic acids (methyl gallate, caffeic, ferulic, gallic and vanillic acids) | **-** | NS |
| Syzygium aromaticum flower (Clove) [[9](#_ENREF_9), [24](#_ENREF_24)] | Eugenyl acetate, eugenol, and β-caryophyllene, flavonoids (kaempferol, quercetin and its derivates) | **+** | NS |
| Humulus lupulus flower (Hop) [[25](#_ENREF_25), [26](#_ENREF_26)] | Flavanones, Flavonols, Flavan-3-ols, Tannins, Phenolic acids, Chalcones | **+** | NS |
| Zingiber officinale  rizhome (Ginger) [[27-29](#_ENREF_27)] | Flavonoids (Luteolin7-glucoside, luteolin, kaempferitrin, rutin, quercetin, naringenin, apigenin, catechin, epicatechin), coumarin, anthocyanins, phenolic acid (gallic, protocatechuic, chlorogenic, caffeic, vanillic, ferulic and cinnamic acids) | NS | **-** |
| Euclea natalensis root (Natal guarri) [[30](#_ENREF_30), [31](#_ENREF_31)] | naphthoquinones, tannins, pentacyclic terpenoids | Enamel **-**  Dentin **+** | NS |
| **Seed** |  |  |  |
| Citrus Grandis (Grapefruit) seed [[2](#_ENREF_2), [7](#_ENREF_7), [32](#_ENREF_32)] | High content of flavonoids (naringenin, hesperidin), phenolic acids (gallic, protocatechuic, p-hydroxybenzoic, vanillic, caffeic, chlorogenic, p-coumaric, ferulic, chlorogenic acids) | **+** | **-** |
| Vitis vinifera ﻿L. (Grape) seed [[1](#_ENREF_1), [2](#_ENREF_2), [6](#_ENREF_6), [7](#_ENREF_7), [33](#_ENREF_33)] | Oligomeric proanthocyanidins (catechin, epicatechin as procyanidins and prodelphinidins, anthocyanins, phenolic acids | Enamel **+**  Dentin **-** | Enamel **-**Dentin **+** |
| Caesalpinia ferrea (Juca seed galactomannan) [[34-36](#_ENREF_34)] | Hydrophilic polysaccharide, phenolic composition (methyl gallate, ellagic acid (*) | NS | **-** |
| **Tea** |  |  |  |
| Camellia sinensis (Green tea) [[7](#_ENREF_7), [8](#_ENREF_8), [37-45](#_ENREF_37)] | Phenolic acids (gallic, chlorogenic, caffeic, p-coumaric and quinic acids, tea gallate), flavonoids (myricetin glycosides, quercetin glycosides, behenyl glycosides), kaempferol, quercetin, anthocyanins (is not high), theaflavins, tannins, catechins (catechin, epicatechin, epigallocatechin, epicatechin gallate, epigallocatechin Gallate) | **+** | **+** |
| Camellia sinensis assamica (Black Tea) [[7](#_ENREF_7), [39](#_ENREF_39), [40](#_ENREF_40), [46](#_ENREF_46), [47](#_ENREF_47)] | Phenolic acids ( gallic and chlorogenic acids**,** theogallin), epigallocatechin gallate, theaflavins, thearubigins, amino acid L-theanine, and other catechins or flavonoids | **+** | **+** |
| **Lipophilic components** |  |  |  |
| Olea europaea (Olive) oil [[48-50](#_ENREF_48)] | Cinnamic and benzoic acids, phenolic alcohols, secoiridoids, lignans, hydroxy-isochromans, flavonoids, monounsaturated and polyunsaturated fatty acids (oleic/linoleic acids) | Enamel (**+**/**-**)  Dentin **-** | NS |
| Arecaceae  (Palm) oil [[49](#_ENREF_49), [51-53](#_ENREF_51)] | Polyphenols (gallic and caffeoylshikimic acids), saturated, monounsaturated and polyunsaturated fatty acids (myristic, palmitic, stearic, oleic/linoleic acids) | **+** | **+** |
| Cocos nucifera (Coconut) oil [[49](#_ENREF_49), [54](#_ENREF_54)] | Phenolic compounds (caffeic, p-Coumaric, ferulic and syringic acids, catechin), saturated and unsaturated fats, high source of lauric acid | **-** | NS |
| Carthamus tinctorius (Safflower) oil [[49](#_ENREF_49), [55](#_ENREF_55)] | Flavonoids (kaempferol, hyperoside, naringenin, quercetin, luteolin), high polyunsaturated fatty acid (linoleic acid) | **-** | NS |
| Helianthus annuus (Sunflower) oil [[49](#_ENREF_49), [56](#_ENREF_56)] | Flavonoids (heliannone, quercetin, kaempferol, luteolin, apigenin), phenolic acids (caffeic, chlorogenic, caffeoylquinic, gallic, ferulic, and sinapic acids, protocatechuic, coumaric), saturated and unsaturated fatty acids (oleic/linoleic acid) | **-** | NS |
| Mentha piperita L. (Peppermint leaves) oil [[7](#_ENREF_7)] | Mainly flavones and flavanones, phenolic acids, (caffeic, ferulic, chlorogenic, gallic and vanillic acids) | **-** | NS |
| **Others** |  |  |  |
| Quercetin [[57-59](#_ENREF_57)] | Quercetin is a plant-derived compound that has different common names depending on the source and context. It is also known as pentahydroxyflavone, quercetine, Vitamin P, indian gooseberry, polyphenolic flavonoid, phytochemical, polyphemol or flavonoid | **+** | **+** |
| Epigallocatechin gallate [[40](#_ENREF_40), [42](#_ENREF_42), [59-62](#_ENREF_59)] | The ester of epigallocatechin and gallic acid, and is a type of catechin that obtained from green tea | **+** | **+** |
| Theaflavin gallate [[40](#_ENREF_40)] | Theaflavin gallate are formed by the oxidation of epicatechin and epigallocatechin-3-gallate in presence of polyphenol oxidase and peroxidase enzymes | NS | **+** |
| Proanthocyanidin [[53](#_ENREF_53), [63](#_ENREF_63)] | The condensed tannins (known as pro-anthocyanidins) are derivatives of flavanol. Flavanols are a specific class of compounds within the much larger family of polyphenolic compounds known as flavonoids | NS | **+**  at pH=7.0 |
| Anacardic acid [[62](#_ENREF_62)] | Anacardic acid is the phenolic lipids from the shell of the cashew nut | NS | **+** |
| Tannic acid [[26](#_ENREF_26), [47](#_ENREF_47), [64](#_ENREF_64), [65](#_ENREF_65)] | Tannic acid is a type of polyphenol and a specific kind of hydrolysable tannin composed of 8 to 10 molecules of gallic acid | **+** | NS |
| Xylitol [[65](#_ENREF_65)] | Xylitol is one of the naturally occurring pentitols (five-carbon sugar alcohol) with a molecular formula of C_5_H_12_O_5_ | **+** | NS |
| Propolis (bee glue) [[7](#_ENREF_7), [66](#_ENREF_66)] | Flavonoids (catechin, luteolin, naringenin, quercetin, kaempferol, lslapinin, ermanin, pectolinarigenin, sakuranetin, Isosakuranetin), phenolic acids (cinnamic and caffeic acids) | **-** | NS |

* Plant extracts that had a positive role in reducing erosion are shown with a (+) sign. Those that did not play a role in reducing erosion or worsened erosion are shown with a (-) sign. NS indicates that the therapeutic or preventive effects of the extract were not investigated.

**Supplementary References**

1. Niemeyer SH, Baumann T, Lussi A, Scaramucci T, Carvalho TS. Plant extracts have dual mechanism on the protection against dentine erosion: action on the dentine substrate and modification of the salivary pellicle. Sci Rep. 2023;13(1):7089.

2. Carvalho TS, Pham K, Rios D, Niemeyer S, Baumann T. Synergistic effect between plant extracts and fluoride to protect against enamel erosion: An in vitro study. PLoS One. 2022;17(11):e0277552.

3. Maya-Cano DA, Arango-Varela S, Santa-Gonzalez GA. Phenolic compounds of blueberries (Vaccinium spp) as a protective strategy against skin cell damage induced by ROS: A review of antioxidant potential and antiproliferative capacity. Heliyon. 2021;7(2):e06297.

4. Niemeyer SH, Jovanovic N, Sezer S, Wittwer LS, Baumann T, Saads Carvalho T. Dual protective effect of the association of plant extracts and fluoride against dentine erosion: In the presence and absence of salivary pellicle. PLoS One. 2023;18(5):e0285931.

5. Nemzer BV, Al-Taher F, Yashin A, Revelsky I, Yashin Y. Cranberry: chemical composition, antioxidant activity and impact on human health: overview. Molecules. 2022;27(5):1503.

6. Baumann T, Niemeyer SH, Lussi A, Scaramucci T, Carvalho TS. Rinsing solutions containing natural extracts and fluoride prevent enamel erosion in vitro. J Appl Oral Sci. 2023;31:e20230108.

7. Niemeyer SH, Baumann T, Lussi A, Meyer-Lueckel H, Scaramucci T, Carvalho TS. Salivary pellicle modification with polyphenol-rich teas and natural extracts to improve protection against dental erosion. J Dent. 2021;105:103567.

8. Kato MT, Cardoso CAB, Jordão MC, Galvão RPO, Iscuissati AGS, Kinoshita AMO, et al. Effect of the cranberry (Vaccinium macrocarpon) juice on reducing dentin erosion: an in vitro study. Braz Oral Res. 2022;36:e076.

9. Sarialioglu Gungor A, Donmez N. Dentin erosion preventive effects of various plant extracts: An in vitro atomic force microscopy, scanning electron microscopy, and nanoindentation study. Microsc Res Tech. 2021;84(5):1042-52.

10. Maphetu N, Unuofin JO, Masuku NP, Olisah C, Lebelo SL. Medicinal uses, pharmacological activities, phytochemistry, and the molecular mechanisms of Punica granatum L.(pomegranate) plant extracts: A review. Biomed Pharmacother. 2022;153:113256.

11. Laurindo LF, Barbalho SM, Araújo AC, Guiguer EL, Mondal A, Bachtel G, et al. Açaí (*Euterpe oleracea* Mart.) in health and disease: A critical review. Nutrients. 2023;15(4):989.

12. Weber MT, Hannig M, Pötschke S, Höhne F, Hannig C. Application of plant extracts for the prevention of dental erosion: an in situ/in vitro study. Caries Res. 2015;49(5):477-87.

13. Cortez RE, Gonzalez de Mejia E. Blackcurrants (Ribes nigrum): A review on chemistry, processing, and health benefits. J Food Sci. 2019;84(9):2387-401.

14. Obeid RF, Ammar MM, Younis SH. Dentinomimetics and cementomimetics of Moringa oleifera leaves extract. Sci Rep. 2023;13(1):19243.

15. Saini RK, Sivanesan I, Keum YS. Phytochemicals of Moringa oleifera: a review of their nutritional, therapeutic and industrial significance. 3 Biotech. 2016;6(2):203.

16. Pop OL, Kerezsi AD, Ciont Nagy C. A comprehensive review of Moringa oleifera bioactive compounds—cytotoxicity evaluation and their encapsulation. Foods. 2022;11(23):3787.

17. Ouari S, Benzidane N. Chemical composition, biological activities, and molecular mechanism of Inula viscosa (L.) bioactive compounds: a review. Naunyn Schmiedebergs Arch Pharmacol. 2024;397(6):3857-65.

18. Hertel S, Graffy L, Pötschke S, Basche S, Al-Ahmad A, Hoth-Hannig W, et al. Effect of Inula viscosa on the pellicle’s protective properties and initial bioadhesion in-situ. Arch Oral Biol. 2016;71:87-96.

19. Karygianni L, Cecere M, Skaltsounis AL, Argyropoulou A, Hellwig E, Aligiannis N, et al. High‐level antimicrobial efficacy of representative Mediterranean natural plant extracts against oral microorganisms. Biomed Res Int. 2014;2014(1):839019.

20. Djakpo O, Yao W. Rhus chinensis and Galla Chinensis–folklore to modern evidence. Phytother Res. 2010;24(12):1739-47.

21. Ren YY, Zhang XR, Li TN, Zeng YJ, Wang J, Huang QW. Galla Chinensis, a Traditional Chinese Medicine: Comprehensive review of botany, traditional uses, chemical composition, pharmacology and toxicology. J Ethnopharmacol. 2021;278:114247.

22. Zhang LL, Li JY, Zhou XD, Cui FZ, Wei L. Chemical and crystallographic study of remineralized surface on initial carious enamel treated with Galla chinensis. Scanning. 2009;31(6):236-45.

23. Ayati Z, Amiri MS, Ramezani M, Delshad E, Sahebkar A, Emami SA. Phytochemistry, traditional uses and pharmacological profile of rose hip: A review. Curr Pharm Des. 2018;24(35):4101-24.

24. Cortés-Rojas DF, de Souza CRF, Oliveira WP. Clove (Syzygium aromaticum): a precious spice. Asian Pac J Trop Biomed. 2014;4(2):90-6.

25. Muzykiewicz A, Nowak A, Zielonka-Brzezicka J, Florkowska K, Duchnik W, Klimowicz A. Comparison of antioxidant activity of extracts of hop leaves harvested in different years. Herba Polonica. 2019;65(3):1-9.

26. Schestakow A, Rasputnis W, Hannig M. Effect of Polyphenols on the Ultrastructure of the Dentin Pellicle and Subsequent Erosion. Caries Res. 2024;58(2):77-85.

27. Aleem M, Khan MI, Shakshaz FA, Akbari N, Anwar D. Botany, phytochemistry and antimicrobial activity of ginger (Zingiber officinale): A review. Int J Herb Med. 2020;8(6):36-49.

28. Ajayi OB, Akomolafe SF, Akinyemi FT. Food value of two varieties of ginger (Zingiber officinale) commonly consumed in Nigeria. ISRN Nutr. 2013;2013(1):359727.

29. Celik Z, Yavlal G, Yanıkoglu F, Kargul B, Tagtekin D, Stookey G, et al. Do ginger extract, natural honey and bitter chocolate remineralize enamel surface as fluoride toothpastes? an in-vitro study. 2021.

30. Maroyi A. Review of ethnomedicinal uses, phytochemistry and pharmacological properties of Euclea natalensis A. DC. Molecules. 2017;22(12):2128.

31. Sales-Peres SH, Xavier CN, Mapengo MA, Forim MR, Silva MdeF, Sales-Peres A. Erosion and abrasion-inhibiting in situ effect of the Euclea natalensis plant of African regions. Braz Oral Res. 2016;30(1):S1806-83242016000100270.

32. Uckoo RM, Jayaprakasha GK, Balasubramaniam VM, Patil BS. Grapefruit (Citrus paradisi Macfad) phytochemicals composition is modulated by household processing techniques. J Food Sci. 2012;77(9):C921-C6.

33. Ma ZF, Zhang H. Phytochemical constituents, health benefits, and industrial applications of grape seeds: A mini-review. Antioxidants (Basel). 2017;6(3):71.

34. Macêdo NS, Silveira ZS, Bezerra AH, Costa JGMD, Coutinho HDM, Romano B, et al. Caesalpinia ferrea C. Mart.(Fabaceae) phytochemistry, ethnobotany, and bioactivities: a review. Molecules. 2020;25(17):3831.

35. De Souza CF, Lucyszyn N, Ferraz FA, Sierakowski MR. Caesalpinia ferrea var. ferrea seeds as a new source of partially substituted galactomannan. Carbohydr Polym. 2010;82(3):641-7.

36. Rabelo CS, Oliveira JMRd, Leal IdC, Costa FdMLL, Ricardo NMPS, Passos VF. The potential of galactomannan from Caesalpinia ferrea on erosive dentin wear reduction in vitro. Braz Dent J. 2023;34(5):72-8.

37. Zhao T, Li C, Wang S, Song X. Green tea (Camellia sinensis): A review of its phytochemistry, pharmacology, and toxicology. Molecules. 2022;27(12):3909.

38. Wang C, Han J, Pu Y, Wang X. Tea (Camellia sinensis): a review of nutritional composition, potential applications, and Omics Research. Applied Sciences. 2022;12(12):5874.

39. Ozan G, Sar Sancakli H, Yucel T. Effect of black tea and matrix metalloproteinase inhibitors on eroded dentin in situ. Microsc Res Tech. 2020;83(7):834-42.

40. Passos VF, Melo MAS, Lima JPM, Marçal FF, Costa CAGA, Rodrigues LKA, et al. Active compounds and derivatives of camellia sinensis responding to erosive attacks on dentin. Braz Oral Res. 2018;32:e40.

41. Mirkarimi M, Toomarian L. Effect of green tea extract on the treatment of dentin erosion: an in vitro study. J Dent (Tehran). 2012;9(4):224-8.

42. DE Mores MDR, Passos VF, Padovani GC, BEZERRA LCBR, Vasconcelos IM, Santiago SL. Protective effect of green tea catechins on eroded human dentin: an in vitro/in situ study. Braz Oral Res. 2021;35:e108.

43. De Moraes MD, Carneiro JR, Passos VF, Santiago SL. Effect of green tea as a protective measure against dental erosion in coronary dentine. Braz Oral Res. 2016;30:S1806-83242016000100213.

44. Kato MT, Magalhães AC, Rios D, Hannas AR, Attin T, Buzalaf MA. Protective effect of green tea on dentin erosion and abrasion. J Appl Oral Sci. 2009;17:560-4.

45. Magalhães AC, Wiegand A, Rios D, Hannas A, Attin T, Buzalaf MA. Chlorhexidine and green tea extract reduce dentin erosion and abrasion in situ. J Dent. 2009;37(12):994-8.

46. Rasheed Z. Molecular evidences of health benefits of drinking black tea. Int J Health Sci (Qassim). 2019;13(3):1-3.

47. Schestakow A, Nekrashevych Y, Hoth-Hannig W, Hannig M. Influence of periodic polyphenol treatment on the anti-erosive potential of the acquired enamel pellicle—a qualitative exploratory study. J Dent. 2022;124:104236.

48. Gouvinhas I, Machado N, Sobreira C, Domínguez-Perles R, Gomes S, Rosa E, et al. Critical Review on the Significance of Olive Phytochemicals in Plant Physiology and Human Health. Molecules. 2017;22(11):1986.

49. Ionta FQ, Alencar CRB, Val PP, Boteon AP, Jordão MC, Honório HM, et al. Effect of vegetable oils applied over acquired enamel pellicle on initial erosion. J Appl Oral Sci. 2017;25(4):420-6.

50. Wiegand A, Gutsche M, Attin T. Effect of olive oil and an olive-oil-containing fluoridated mouthrinse on enamel and dentin erosion in vitro. Acta Odontol Scand. 2007;65(6):357-61.

51. Abdullah F, Ismail R, Ghazali R, Idris Z. Total phenolic contents and antioxidant activity of palm oils and palm kernel oils at various refining processes. Journal of Oil Palm Research. 2018;30(4):682–92.

52. Ionta FQ, Alencar CRB, Santos NMD, Bergantin BTP, Val PP, Honório HM, et al. Effect of palm oil alone or associated to stannous solution on enamel erosive-abrasive wear: A randomized in situ/ex vivo study. Arch Oral Biol. 2018;95:68-73.

53. Martins DS, Boteon AP, Ferreira AM, Debortolli ALB, Grizzo IC, Ionta FQ, et al. Can the combination of proanthocyanidin and vitamin E or palm oil effectively protect enamel against in vitro erosive and abrasive challenges? J Appl Oral Sci. 2024;32:e20240100.

54. Seneviratne KN, Sudarshana Dissanayake DM. Variation of phenolic content in coconut oil extracted by two conventional methods. International journal of food science & technology. 2008;43(4):597-602.

55. Hannig C, Wagenschwanz C, Pötschke S, Kümmerer K, Kensche A, Hoth-Hannig W, et al. Effect of safflower oil on the protective properties of the in situ formed salivary pellicle. Caries Res. 2012;46(5):496-506.

56. Guo S, Ge Y, Na Jom K. A review of phytochemistry, metabolite changes, and medicinal uses of the common sunflower seed and sprouts (Helianthus annuus L.). Chem Cent J. 2017;11(1):95.

57. Capalbo LC, Delbem ACB, Dal-Fabbro R, Inácio KK, de Oliveira RC, Pessan JP. Effect of sodium hexametaphosphate and quercetin, associated or not with fluoride, on dentin erosion in vitro. Arch Oral Biol. 2022;143:105541.

58. Li XY, Lin XJ, Zhong BJ, Yu H. Effects of the application timing of anti-erosive agents on dentin erosion. J Mech Behav Biomed Mater. 2022;136:105512.

59. Jiang NW, Hong DW, Attin T, Cheng H, Yu H. Quercetin reduces erosive dentin wear: evidence from laboratory and clinical studies. Dent Mater. 2020;36(11):1430-6.

60. Iftikhar A, Zafar S, Khalil SK, Awais SM, Batool S, Shahid M. The Effect of Epigallocatechin Gallate Extract on Dental Erosion an in Vitro Study. Pakistan Journal of Medical & Health Sciences. 2022;16(09):610-.

61. Wang YL, Chang HH, Chiang YC, Lu YC, Lin CP. Effects of fluoride and epigallocatechin gallate on soft-drink-induced dental erosion of enamel and root dentin. J Formos Med Assoc 2018;117(4):276-82.

62. Silveira C, Oliveira F, Dos Santos ML, De Freitas T, Imparato JC, Magalhães AC. Anacardic acid from brazilian cashew nut trees reduces dentine erosion. Caries Res. 2014;48(6):549-56.

63. Cardoso F, Boteon AP, Silva TAPd, Prakki A, Wang L, HonÓrio HM. In situ effect of a proanthocyanidin mouthrinse on dentin subjected to erosion. J Appl Oral Sci. 2020;28:e20200051.

64. Hertel S, Pötschke S, Basche S, Delius J, Hoth-Hannig W, Hannig M, et al. Effect of Tannic Acid on the Protective Properties of the in situ Formed Pellicle. Caries Res. 2017;51(1):34-45.

65. Souza JG, Rochel ID, Pereira AF, Silva TC, Rios D, Machado MAA, et al. Effects of experimental xylitol varnishes and solutions on bovine enamel erosion in vitro. J Oral Sci. 2010;52(4):553-9.

66. Šturm L, Ulrih NP. Advances in the propolis chemical composition between 2013 and 2018: A review. Efood. 2020;1(1):24-37.
